# Biogenic amine tryptamine in human vaginal probiotic isolates mediates matrix inhibition and thwarts uropathogenic *E. coli* biofilm

**DOI:** 10.1038/s41598-024-65780-0

**Published:** 2024-07-04

**Authors:** Veena G. Nair, C. S. Srinandan, Y. B. R. D. Rajesh, Dhiviya Narbhavi, A. Anupriya, N. Prabhusaran, Saisubramanian Nagarajan

**Affiliations:** 1 Microbial Biofilm Lab, Centre for Research in Infectious Diseases, School of Chemical and Biotechnology, SASTRA Deemed to be University, Thanjavur, Tamil Nadu 613401 India; 2 Department of Chemistry, School of Chemical and Biotechnology, SASTRA Deemed University, Thanjavur, Tamil Nadu 613 401 India; 3 Department of Obstetrics and Gynaecology, TSRMMCH&RC, Tiruchirappalli, Tamil Nadu India; 4 Department of Microbiology, TSRMMCH&RC, Tiruchirappalli, Tamil Nadu India; 5 Research Faculty, Institutional Research Board TSRMMCH&RC, Tiruchirappalli, Tamil Nadu India; 6 Antimicrobial Resistance Lab, Centre for Research in Infectious Diseases, School of Chemical and Biotechnology, SASTRA Deemed to be University, Thanjavur, Tamil Nadu 613401 India

**Keywords:** Human vaginal probiotics, Cell free supernatant, Tryptamine, Colonization resistance, Matrix inhibition, Antibiofilm, Competitive inhibition, Clinical microbiology, Drug discovery

## Abstract

Probiotics offer a promising prophylactic approach against various pathogens and represent an alternative strategy to combat biofilm-related infections. In this study, we isolated vaginal commensal microbiota from 54 healthy Indian women to investigate their probiotic traits. We primarily explored the ability of cell-free supernatant (CFS) from *Lactobacilli* to prevent Uropathogenic *Escherichia coli* (UPEC) colonization and biofilm formation. Our findings revealed that CFS effectively reduced UPEC’s swimming and swarming motility, decreased cell surface hydrophobicity, and hindered matrix production by downregulating specific genes (*fimA, fimH, papG,* and *csgA*). Subsequent GC–MS analysis identified Tryptamine, a monoamine compound, as the potent bioactive substance from *Lactobacilli* CFS, inhibiting UPEC biofilms with an MBIC of 4 µg/ml and an MBEC of 8 µg/ml. Tryptamine induced significant changes in *E. coli* colony biofilm morphology, transitioning from the Red, Dry, and Rough (RDAR) to the Smooth and White phenotype, indicating reduced extracellular matrix production. Biofilm time-kill assays demonstrated a four-log reduction in UPEC viability when treated with Tryptamine, highlighting its potent antibacterial properties, comparable to CFS treatment. Biofilm ROS assays indicated a significant elevation in ROS generation within UPEC biofilms, suggesting a potential antibacterial mechanism. Gene expression studies with Tryptamine-treated samples showed a reduction in expression of curli gene (*csgA*), consistent with CFS treatment. This study underscores the potential of Tryptamine from probiotic *Lactobacilli* CFS as a promising antibiofilm agent against UPEC biofilms.

## Introduction

Bacteria mostly live in biofilms under natural habitat^1^. An organised group of microbial cells residing in a matrix rich in polymers is known as a biofilm^2^. The biofilm’s polymeric matrix acts as a barrier, shielding the cells from harm such as chemical stressors, radiation, dehydration, and predation while also boosting their tolerance to antimicrobial agents and immunological responses. Matrix formation additionally benefits biofilm cells with increased nutrition availability and other societal advantages^3^. Urinary tract infections (UTIs) are common bacterial infections affecting millions of individuals worldwide. The conventional treatment for UTIs involves the use of antibiotics to eliminate the infecting microorganisms. However, the overreliance on antibiotics has led to the emergence of antibiotic resistance among uropathogens, rendering traditional treatment options less effective^4^. The main etiological causes of urinary tract infections (UTIs) are uropathogenic *Escherichia coli* (UPEC). The recurrence of these infections (rUTIs) is a common and challenging problem that frequently results in significant morbidity^5^. The pathogenesis of UPEC is greatly influenced by biofilm (intracellular bacterial communities), which results in the infection’s persistence and recurrence^6^. Additionally, the development of biofilms on urinary catheters represents a significant global problem, accounting for 40% of hospital-acquired infections and offering significant treatment hurdles^7^. The two most important matrix components in UPEC are curli and cellulose. The amyloid protein Curli is a key player in stimulating adhesion, contacts with surfaces, and functions as a structural support for the biofilms^8^. On the other hand, cellulose provides flexibility, architecture, and helps in spatial organization within the biofilm^9^.

The repeated use of antibiotics to treat recurrent UTI infections can promote the survival and proliferation of antibiotic-resistant strains of uropathogens, making subsequent treatment regimens less effective^10^. In recent years, there has been a growing recognition of the limitations of antibiotic-based approaches and a shift towards antibiotic-sparring strategies for the management of UTIs, especially in cases of recurrent infections^11,12^. By utilizing non-antibiotic-based interventions, such as probiotics or other novel therapeutic agents like mannosides and pilicides^13^ that prevent uropathogens from attaching to bladder epithelial cells, such interventions can possibly eliminate them and restore the balance of the urinary tract microbiota, while minimizing the adverse effects on the host microbial community. Considering the global trend towards antibiotic-free treatments for recurrent UTIs, it is essential to explore the efficacy of these alternative approaches, understand their mechanisms of action, safety profiles, and evaluate its potential to prevent recurrent UTIs while minimizing the risk of antibiotic resistance development.

Studies on probiotics have effectively evolved over time, with the more recent ones showing credible evidence that probiotic bacteria can enhance human health^14,15^. *Lactobacilli* accounted for about 70% of the total culturable bacteria isolated from healthy women^16^. In the vaginal microbiome of healthy women of reproductive age, *Lactobacilli* play a crucial role, notably in preserving homeostasis and avoiding urogenital infection. *Lactobacilli* can synthesise organic acids, bacteriocins, hydrogen peroxide, and bacteriocin-like inhibitory compounds. The cell free supernatant (CFS) of *Lactobacillus* strains isolated from healthy women developed antagonistic activities against *Gardnerella vaginalis* and *Prevotella bivia*, which are associated with bacterial vaginosis^17^. CFS of *Lactobacilli* inhibits the growth of multiple bacterial pathogens, including ESBL-producing *Klebsiella pneumoniae* and *Pseudomonas aeruginosa*, carbapenem-resistant Enterobacteriaceae and other pathogens like *Listeria monocytogenes*, *Staphylococcus aureus*, *Salmonella* Typhimurium, and *Clostridium difficile*^18^. Other report has shown that the CFS of *Lactobacillus casei* had anti-Shigella activities *in vitro*^19^. Studies have shown that *Lactobacillus* CFS exhibited antibacterial activities against a wide range of food borne pathogens, including *Listeria monocytogens*, *Staphylococcus aureus*, *Salmonella* Typhimurium and *E. coli* O157:H7, *E. cloacae*, *E. faecalis* and *Clostridium difficile, H. pylori, Campylobacter jejuni*^20–22^. Irrespective of the source of *Lactobacilli*, CFS preparations from 46 lactic acid bacteria isolated from different raw and fermented milk products exhibited antibacterial activities against *Enterococcus faecalis, E. coli, Salmonella spp, Shigella sonnei, Staphylococcus aureus*, Methicillin Resistant *Staphylococcus aureus* (MRSA), and *Listeria monocytogenes*^23^.

In the present study we screened and characterized the metabolites in the cell-free supernatant that actively contributes to the inhibition of biofilm matrix in UPEC and identified Tryptamine, a biogenic amine, from the cell free supernatant of *Lactobacillus*, exhibits strong antibiofilm potential and inhibits the formation of biofilm matrix in Uropathogenic *E. coli* biofilm. Tryptamine is produced by several species of gut bacteria, primarily from the genera *Clostridium, Ruminococcus, Blautia, and Bacteroides.* Tryptamine itself may provide certain bacteria with a survival advantage, as it contributes to acid stress tolerance in *R. gnavus.* The production of tryptamine by these gut bacteria is dependent on the enzyme tryptophan decarboxylase (TDC), which catalyzes the conversion of tryptophan to tryptamine^24–26^. Tryptamine derivatives have emerged as powerful antibacterial agents capable of neutralizing colistin resistance in polymyxin-resistant Gram-negative bacteria. These compounds have demonstrated effectiveness in reinstating the potency of colistin against resistant strains^27^. Tryptamine derivatives exert their antibacterial effect by interacting with the bacterial cell membrane, causing disruption and eventual cell death. This mode of action differs from conventional antibiotics, which typically target specific bacterial enzymes or proteins^28^. Tryptamine showed promising antimicrobial activity against *Vibrio carchariae* with a minimum inhibitory concentration (MIC) of 1 µg/ml^29^. The antimicrobial activity of tryptamine-based peptoids were compared with that of ciprofloxacin wherein, all new peptoids displayed antimicrobial activity, with some displaying higher efficacy than ciprofloxacin^30^. Despite the knowledge about of antibacterial effects of tryptamine, its anti-biofilm properties are sparsely reported. The exploration of alternative strategies like exploiting the antibiofilm properties of tryptamine offers promising avenues for managing UTIs and combating antimicrobial resistance by effectively curtailing the formation of UPEC biofilms.

## Materials and methods

### Target population

Fifty-four healthy South Indian women aged 18–40 years who visited the outpatient clinic with healthy vaginal mucosa at the Obstetrics and Gynaecology Department, Trichy SRM Medical College and Research Centre between June 2022 and July 2022 had consented to participate in the study. Informed consent was obtained from all study participants before they were enrolled. Vaginal swabs were collected from 54 healthy participants who were enrolled. The sampling methods were carried out in accordance with the Human Ethical Committee’s recommendations on human testing (institutional and national). The ethics committee authorised the study protocol (Registration number: 696/TSRMMCH& RC/ME-1/2022-IEC No: 127).

### Exclusion and inclusion criteria

Volunteers who were on antibiotics, probiotics, immune suppressants, or hormone treatments, as well as pregnancy or breastfeeding, were all excluded, as were clinically obvious, sexually transmitted viral and bacterial infections such as Human papillomavirus infection*, Chlamydia* spp. infections, Syphilis, Gonorrhoea, and bacterial vaginosis (BV), as well as parasitic infections such as *Trichomonas vaginalis* infection. Preclinical data on co-morbidities, method of delivery, menstrual state, hormone medication, antibiotic administration, contraceptive usage, and clinical information such as the history of urinary tract infection (UTI), Diabetes, hypertension, and other clinical manifestations were evaluated.

### Identification of bacterial strains

All vaginal swabs were collected from healthy women from the upper third part of the lateral vaginal wall using HIMEDIA-M HI Culture™ Transport Swabs w/Amies Medium w/o Charcoal. The swabs were inoculated onto MRS agar (de Man-Rogosa Sharpe Agar, HI media, India) and the plates were incubated at 37 °C for 48 h, both micro aerobically and anaerobically. To achieve pure cultures, discrete colonies were selected and subcultured. Gram stain and colony morphology were used to identify* Lactobacilli* at the genus level. Genomic DNA was isolated from Lactobacillus isolates according to the manufacturer’s instructions using the QIAamp DNA micro kit (Qiagen, Valencia, CA, USA) and used as a template for amplification. Sequencing of the 16S rRNA was performed using 0.1 mM forward: 5′ AGA GTT TGA TCC TGG CTC AG and 3′ reverse: 5′ CCC ACT GCT GCC TCC CGT AG 3′ primers. The PCR was carried out in a 50 µl volume comprising 5 µl 10 × buffer, 2.5 mM MgCl_2_, 200 mM each dNTP, 10 pmol of each primer, 0.5 U Taq polymerase, and 5 µl of Lactobacilli DNA, with the remainder volume made up with sterile distilled water. Amplification commenced with a 3-min denaturation at 95 °C, with 30 cycles of 1 min at 95 °C, 1 min at 48 °C, 2 min at 72 °C, and 10 min at 72 °C. The PCR products were purified using the QIA quick PCR purification kit (Qiagen Valencia, CA, USA) and subsequently analysed with the same primers using the Applied Biosystems 3730xl DNA Analyzer, Catalog number: 3730XL and Dye Terminator Chemistry. The sequences were retrieved using a blast search, and the genus and species were determined using the threshold of 95 percent sequence similarity for genus and species. The phylogenetic tree was constructed using Mega 11 software.

### Biofilm development assay

The biofilm development of *Lactobacilli* strains was evaluated as described previously by Miryala et al.^31^ with minor modifications. In a 96-well polystyrene microtiter plate, 200 μl of test medium was added to each well. 20 μl of culture grown in MRS broth for 48 h was used as the inoculum and the inoculated culture was incubated at 37 °C at 48 h in static conditions. To determine the extent of biofilm formation, the broth was decanted, and the unbound cells were washed thrice with sterile Phosphate Buffered saline, 1% Crystal Violet (CV) was used to stain the surface-bound cells for 15 min, followed by rinsing twice with 200 μl of distilled water. After air drying, the cell/matrix bound dye was extracted using 70% ethanol and the absorbance were measured at 595 nm in a plate reader (Tecan Sunrise). To ensure the influence on biofilm development wasn’t due to a nonspecific binding effect of crystal violet, sterile media was used as a negative control. Based on three standard deviations above the OD mean value recorded for the negative control, a cut-off OD (ODc) threshold was taken into consideration.

### Coaggregation assay

The coaggregation property was evaluated according to Caggie et al.^32^. *Lactobacilli* strains were grown for 24 h at 37 °C in MRS Broth. The pathogenic strains used were *Klebsiella oxytoca*, *Klebsiella pneumoniae*, *Citrobacter koserii* (isolated from the urine of patient with urinary tract infection), the organism and its antibiotic profile, were identified using VITEK-2 system Uropathogenic *E-coli* UTI89 (gifted by Professor Matthew A. Mulvey, University of Utah) and *E. coli* CFT073 were grown in Luria Bertani (LB) Broth for 24 h at 37 °C. 2 ml each of *Lactobacilli* and pathogenic strain suspensions were mixed and incubated at 37 °C without agitation. After two hours, the absorbance was measured using a plate reader. The control strains used are *L. fermentum* MTCC and *L. rhamnosus* MTCC.

### Hydrophobicity

Microbial adhesion to solvents (MATS) was tested with some modifications from Rosenberg et al.^33^ Briefly, bacteria were isolated from the stationary phase by centrifugation (5,000 g, 15 min), washed twice and resuspended to a concentration of around 10^8^ CFU/ml in phosphate buffer (pH = 7.0). The absorbance of the cell suspension was measured at 600 nm. To 3 ml of cell suspension, one millilitre of xylene was added. The two-phase system was preincubated for 10 min at RT, vortexed for 2 min and allowed to separate. After 2 h of incubation at room temperature, the aqueous phase was removed, and its absorbance at 600 nm was determined.

### Competitive inhibition of vaginal Lactobacilli against urinary pathogens

The experiment was performed according to Miryala et al.^31^, with minor modifications. The urinary pathogens and the *Lactobacilli* strains were cultivated in LB broth and simulated vaginal media (SVM) by overnight incubation at 37 °C for 24 h. 1 ml of *Lactobacilli* culture and 1 ml of pathogenic bacteria both at a equal cell density of 10^7^–10^8^ were added simultaneously to the same well of 24 well plate followed by an incubation of 37 °C for 24 h. After the incubation, non-adhered cells were removed by washing twice with PBS. The adhered cells were scraped out, serially diluted and 100 µl was plated on nutrient agar for the pathogens and on MRS agar for the *Lactobacillus* isolates, The inoculated plates were incubated for 24 h at 37 °C. The normalization of cell counts of mono and formed co-culture was performed. The relative fitness was calculated as reported earlier by Lenski et al.^34^ and computed fitness represented by Malthusian parameter M = ln(N1/N0) was determined. In co-culture experiments, the relative fitness (RM) was computed by taking the ratio of fitness values of pathogen with that of *Lactobacillus* sp.

### Time-kill assay

A time kill analysis was conducted to assess the bactericidal efficacy of a cell-free supernatants (CFS) of Lactobacilli against biofilm cells. Uropathogenic bacteria in the early log phase were allowed to form biofilm, and after 48 h, the supernatant was carefully removed. The biofilm was then rinsed with phosphate-buffered saline (PBS) and treated independently with 50 µl of cell-free supernatants and with MBEC concentration of Tryptamine obtained from *Lactobacillus* cultures as well as with the procured tryptamine. Subsequently, at regular intervals of 2 h up to 24 h, the biofilm was carefully scraped using a rubber policeman. The scraped biofilm was then subjected to serial dilution and plated onto LB agar media. The plated samples were incubated for 24 h at 37 °C. After the incubation period, the colony-forming units (CFUs) of biofilm derived cells were  determined^35^.

### ROS assay

ROS generated due to CFS treatment was determined using reduction of DCFH-DA as reported earlier. Briefly, the preformed biofilm were treated with CFS and with tryptamine, ascorbic acid (100 mM), as ROS inhibitor and a combination with CFS and with tryptamine, the biofilms were scrapped using rubber policeman and resuspended in sterile PBS, followed by the addition of a 10 μM stock solution of DCFH-DA dye. The mixture was incubated for 20 min, centrifuged, and the samples were washed with PBS to remove excess dye. The fluorescence intensity was determined using a fluorescence microplate reader with an excitation of 485 nm and emission of 538 nm. Hydrogen peroxide was used as the positive control^36^.

### Exopolysaccharide (EPS) extraction and quantification of uropathogenic *E. coli* (UPEC UTI89)

To measure EPS production, bacteria were cultured on Durapore membrane filters (0.22 µm GV, Millipore) placed on YESCA agar plates. After cultivation, the bacteria were suspended in MilliQ H_2_O and adjusted to 0.5 OD in 500 µl. The suspension was then heated at 100 °C for 20 min to deactivate potential polysaccharide-degrading enzymes, followed by centrifugation at 20,000*g* for 20 min. The resulting supernatant was collected, and EPS were precipitated using ethanol^37^. The total neutral sugar content in the EPS extracts, expressed as glucose equivalents, was quantified using glucose as a standard^38^.

### Scanning electron microscopy

*Escherichia coli* UTI89 cells in the late exponential phase were allowed to form biofilms on glass slides (1cm x1cm) to obtain samples for scanning electron microscopy (SEM) and then fixed with 2.5% glutaraldehyde solution overnight at 4 °C. After fixation, a series of graded ethanol concentrations (varying from 30 to 100%) were used to dehydrate the cells, followed by washing thrice with PBS (pH 7.4). The desiccated samples were mounted onto aluminium stubs, sputter coated with a gold–palladium layer and examined using a digital field emission scanning electron microscope (JEOL-JSM-670IJ).

### Motility assay

Swim plates containing 0.3% of agar and swarm plates containing 0.7% of agar supplemented with 0.5% of glucose were incorporated with MIC and Sub-MIC concentration of CFS and tryptamine. Briefly, bacteria grown overnight were diluted 1000-fold in LB and incubated at 37 °C to 0.1 OD at 600 nm. Swarm plates were inoculated into the middle of soft agar by spotting with 2 μl of standardized culture. Swim plates were seeded with the same inoculum below the agar surface using a sterile inoculating needle. Plates were incubated for 24 h at 37 °C to discern the impact of CFS metabolites on swimming and swarming motility^39^.

The quantitative measurements for the swarming and swimming motility assays were determined and the diameters of the colonies was determined using ImageJ software.

### Live/dead staining

The BacLight bacterial Viability Kit from Invitrogen was used to ascertain if the culture was alive or dead. Propidium iodide (red) and SYTO9 (green) are used in the kit to distinguish between live and dead cells, respectively. CFS treated and untreated biofilms formed on the glass slide was stained with 3 μl of a 1:1 mixture of SYTO9-PI reagent, subsequently, the slide was incubated in dark for 15 min at room temperature. The Nikon Eclipse Ts2 fluorescent microscope was used for observing the live-dead status of treated/untreated samples.

### Gene expression studies

The test culture petriplates treated with CFS and Tryptamine of *L. jensenii* and the untreated control plate were incubated under static conditions at 37 °C for 24 h. The RNeasy mini kit (Qiagen Technologies, Hilden, Germany) was used in accordance with the manufacturer’s instructions for bacterial RNA extraction. High-capacity cDNA reverse transcription kit (Bio-Rad) was used to synthesise cDNA from high-quality total RNA. The RNA and cDNA were quantified using Nanodrop-One (Thermo Fischer Scientific, Waltham, MA). qRT-PCRs were performed using iQTM SYBR^®^ Green Supermix (Bio-Rad) in a QuantStudio™ 5 System (Applied biosystem, Thermofisher Scientific, Waltham, MA) for biofilm related gene targets. 16S rRNA gene was used as internal control, the gene expression levels were evaluated and expressed as a relative fold-difference using ∆∆Ct method^40^.

### Gas chromatography–mass spectrometry (GC–MS)

Gas chromatography–mass spectrometry (GC–MS) analysis of column chromatography separated fractions were conducted with a Shimadzu QP2010 SE mass detector. The carrier solvent utilized was IJC2 methanol. Operating conditions for GC–MS included an injection temperature of 280.00 °C with a split injection mode and a column oven temperature set at 50.0 °C. The system maintained a pressure of 53.5 kilopascals (kPa) and a total flow rate of 12.0 ml per minute (mL/min), with a column flow rate of 1.00 ml/min. The flow control mode was linear, with a linear velocity of 36.3 cm per second (cm/sec). A purge flow of 3.0 ml/min and a split ratio of 8.0 were employed. Mass spectrometry parameters included a scan speed of 1666, covering the mass-to-charge ratio (m/z) range from 40.00 to 600.00.

### Adhesion, biofilm, quantification of matrix and metabolic viability assay

The quantification of biofilm biomass and viable cells in different stages of *E. coli* UTI89 biofilm formation was conducted using microtiter plates. A 96-well microtiter plate with flat-bottom wells (Tarsons, Kolkata) was utilized for the crystal violet assay. Additionally, a 24-well microtiter plate (Tarsons) was employed to assess viable cells at various biofilm stages. Approximately 10^7^ cells/mL of *E. coli* UTI89, derived from an overnight culture, were inoculated into the microtiter wells containing YESCA broth. These plates were then incubated at 37 °C under static conditions. The adhesion stage was assessed by incubating the *E. coli* UTI89 culture for 1 h. For the dispersal studies, preformed *E. coli* UTI89 biofilms were allowed to develop on the microtiter well surfaces for 24 h. After the respective incubation periods, the liquid medium was removed (decanted), and unbound cells were eliminated by rinsing the wells three times with sterile phosphate-buffered saline (PBS). The cells that had adhered to the surface of the microtiter wells were stained with crystal violet dye (CV) for 15 min. Subsequently, excess CV dye was removed, and the wells were de-stained using 70% ethanol and the measurement of biofilm biomass was determined for the ethanol extracted CV layer at 595 nm using a plate reader (Tecan Sunrise). For determining the number of viable cells, the wells were scraped with a sterile rubber policeman. The collected cells were then subjected to a tenfold dilution series, followed by spread plating. This method enabled the enumeration of colony forming units (CFU) representing viable cells^31^.

We have performed the MTT assay to assess the metabolic viability of the preformed biofilms after treatment with tryptamine. The experiment was conducted using overnight cultures in the log phase at OD600.The preformed biofilm was established, 20 μl of MTT (5 mg/ml) was added to each well and incubated at 37 °C for 2 h. The positive control used was EDTA (0.1%) After discarding the solution, 100 μl DMSO was added and incubated at 37 °C for 20 min. The Optical density was measured at 540 nm using a microplate reader (Tecan Sunrise)^41^.

### Congo-red binding assay

Curli proteins, which constitute a significant component of the *E. coli* UTI89 biofilm matrix, were quantified using congo red binding assay as reported earlier^42^ *E. coli* UTI89 cultures were grown under static conditions for 24 h, with and without commercial tryptamine supplementation. After incubation, the cultures were centrifuged at 13,000 rpm for 2 min to pellet the bacterial cells. The resulting pellet was resuspended in sterile phosphate-buffered saline (PBS) and vortexed to ensure uniform dispersion of cells. The absorbance of the resuspended pellet was recorded at a wavelength of 595 nm. Following absorbance measurement, the resuspended pellet was again subjected to centrifugation. After centrifugation, the pellet was resuspended in a solution of Congo red (CR) dye (0.004% in PBS) and allowed to incubate for 10 min at room temperature. After the incubation period, the sample were centrifuged again at 13,000 rpm for 2 min. The absorbance of the supernatant was measured at a wavelength of 487 nm, and this measurement was compared to a CR solution in PBS. CR binding units were calculated as

$${\text{CR}}\;{\text{binding}}\;{\text{unit}} = {[}CR\;binding\;unit = \frac{{{\Delta }OD\;at\;487\;{\text{nm}}}}{{Culture\;OD\;at\;595\;{\text{nm}}}} \times 100{]}$$

### Calcofluor staining method and Congo red depletion assay

*E. coli* UTI89 and ∆csgA (curli mutant) were spotted onto YESCA Agar supplemented with 2% Calcofluor white stain (Sigma Aldrich). These agar plates were then incubated for a period of 3 days at a temperature of 25 °C. After the incubation period, the colony biofilms that had formed were carefully retrieved from the agar plates. The retrieved colony biofilms were mixed with 1 ml of phosphate-buffered saline (PBS) to remove any unbound cells and excess Calcofluor stain. This mixture was then subjected to centrifugation at 5000 rpm for a duration of 10 min. Following centrifugation, the cells in the biofilm were resuspended in water and transferred to a 96-well microplate (Tarsons). The quantification of biofilm-bound cellulose was performed using fluorescent measurements, with excitation at 360 nm and emission at 460 nm. This measurement was conducted using a Fluorescence Microplate reader (Biotek)^43^.

For the congored depletion assay, two microliters of overnight-grown cultures were spotted in biological triplicates on YESCA Agar plates with and without tryptamine and biofilms were allowed to form for 3 days at 25 °C. After the incubation period, each biofilm colony was carefully scraped from the agar plate. The collected biofilm material was resuspended in 1 ml of PBS containing 40 μg/ml Congo red dye. Subsequently, the samples were incubated at 37 °C for 1 h. Following incubation, the samples were subjected to centrifugation at 16,873×*g* for 2 min. The supernatants obtained after centrifugation were transferred to a clear 96-well plate (Tarsons). The absorbance of these supernatants was then measured at a wavelength of 490 nm using a plate reader (Tecan Infinite F50 ELISA Reader)^44^.

### Ethics approval

The sample collection was carried out with the Human ethical committee approval (Registration number: 696/TSRMMCH& RC/ME-1/2022-IEC No: 127) and all applicable international/national guidelines were followed.

### Informed consent

Informed consents were obtained from the participants before they were enrolled.

## Results

### Isolation, screening, and identification of culturable vaginal probiotic bacteria

The demographic characteristics of participated fifty-four healthy women were surveyed and the details are provided in Table S1. Thirty isolates were obtained from vaginal swabs of fifty-four healthy women’s vaginal ecosystem and among them eight were identified as *Lactobacillus* based on their morphological and genotypic characteristics. The 16srRNA amplification & sequence analysis was performed and the species-level identification of isolated *Lactobacilli* were done. Eight *Lactobacillus* sp. isolated from healthy women were as follows, *Lactobacillus jensenii* (n = 1), *Limosilactobacillus fermentum* (n = 3), *Lactobacillus crispatus* strain (n = 1), *Lactobacillus amylovorus* (n = 1), *Lactobacillus fornicalis* (n = 1), *Weissella confusa* strain (n = 1), *Ligilactobacillus salivarius* (n = 1). In addition, 3 reference *Lactobacilli* strain was included in the present study viz., *Lactobacillus fermentum* MTCC 903, *Lactobacillus rhamnosus* MTCC 1408 and *Lactobacillus acidophilus* MTCC 10,307 respectively.

The phylogenetic tree was constructed using the 16srRNA sequences (Fig. S1). The sequences were submitted to GenBank (https://www.ncbi.nlm.nih.gov/genbank/) and the accession numbers of the isolated strains are provided in (Table S2). A comprehensive list of isolated strains other than *Lactobacillus* sp. were also provided along with their GenBank accession number (Table S3).

### Vaginal *Lactobacilli* competitively inhibits uropathogens

To simulate the in vivo conditions, experiments were performed with MRS and Simulated vaginal medium (SVM) containing mucin. *L. fermentum* had the greatest adhesion percentage of 98% in both MRS and Simulated Vaginal Media The adhesion capacity for all *Lactobacilli* strains in MRS and SVM ranged between 73 and 98% (Fig. S2). Enhanced adhesion in the presence of mucin implies that these *Lactobacilli* strain has a better potential to colonize vaginal epithelium in the presence of mucin than in its absence. Overall, the findings showed that mucin is crucial for adhesion, and biofilm formation and that some strains may be able to utilise it as a carbon source. In short, the isolated *Lactobacillus* sp. has enhanced potential to colonise the vaginal mucosa. Even though, the ability of isolated *Lactobacilli* isolates to adhere, co aggregate UTI pathogens and display enhanced growth in the presence of mucin are desirable features, the potential of the isolated *Lactobacilli* to compete with UTI pathogens in colonization gains importance which would reveal if *Lactobacilli* can indeed prevent UTI pathogens from colonizing. Towards this end, competition experiments between *Lactobacilli* and uropathogens were undertaken.

All strains were tested against the clinically isolated Urinary pathogens which included Multidrug-resistant (MDR) *Klebsiella oxytoca, Klebsiella pneumoniae, Citrobacter koserii, E. coli UTI89* and *E coli* CFT073 (Fig. S3). Our observations showed that all the strains except *L. crispatus* were able to inhibit the most common urinary tract pathogens such as *E. coli UTI89* and *CFT073 *(*p* < 0.01). Nevertheless, there is a strain-specific interaction among the *Lactobacillus* sp. and pathogens*.* For MDR *K. oxytoca, only L. fornicalis and L. salivarius* strains displayed antagonistic interactions (Fig. S3.A). Ability of most *Lactobacilli* strains to inhibit UPEC strains UTI89 and CFT073 which constitute 70% of UTI infections show that *Lactobacilli* strains were highly efficient in preventing colonization by uropathogens.

### Suppression of *E. coli* biofilm matrix by cell free supernatants of isolated vaginal Lactobacilli strains

**Figure 1:**
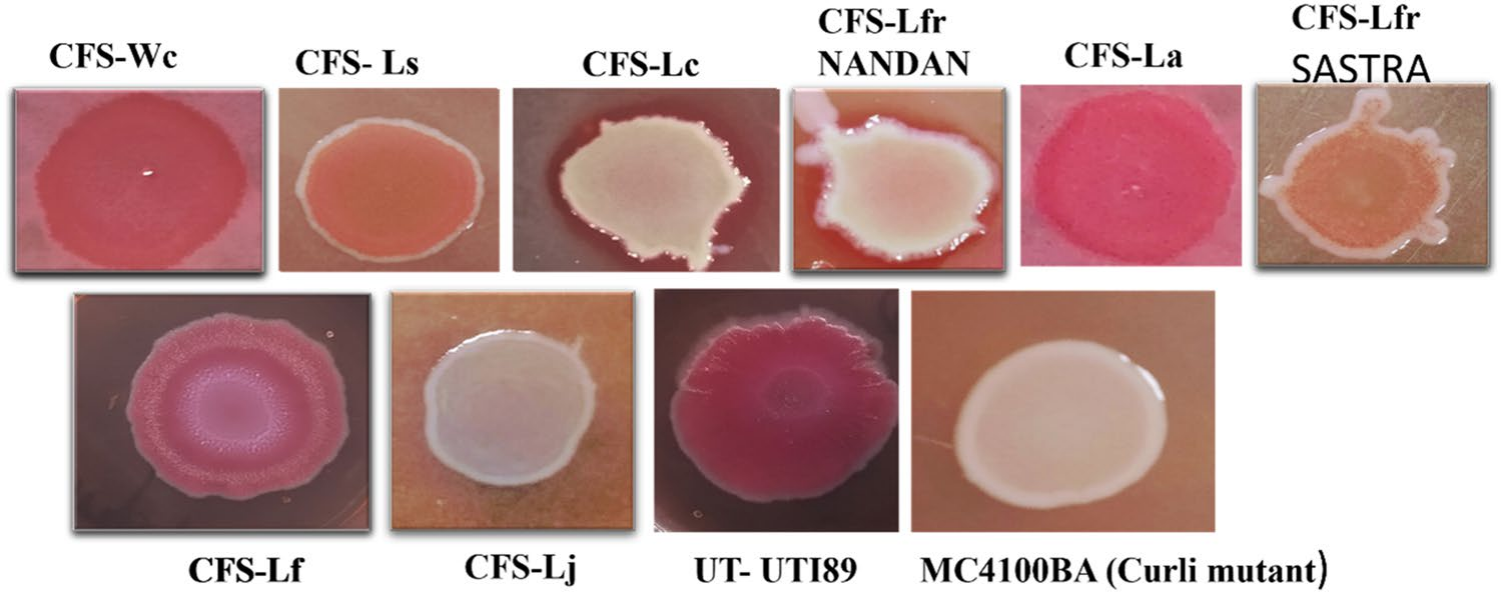
Screening of Cell free supernatant from isolated vaginal probiotics for biofilm matrix inhibition. CFS denotes the cell free supernatant, Wc—*Weissella confusa*, Ls—*L. salivarius*, Lc—*L. crispatus*, L fr NANDAN—*L. fermentum* NANDAN, La—*L. amylovorus*, L fr SASTRA—*L. fermentum* SASTRA, Lf—*L. fornicalis*, Lj—*L. jensenii*, UT-UTI89—Untreated *E. coli* UTI89, MC4100BA- *E. coli* mutant strain which is devoid of curli.

We screened a total of eight cell-free supernatants (CFS) from isolated vaginal probiotics, identifying specific candidates that effectively curbed matrix production in *E. coli* UTI89 (Fig. 1). Red, Dry and Rough (RDAR) colony morphology indicate matrix production whereas Smooth and White (SAW) colony morphology indicates absence of matrix production. Notably, the CFS derived from *L. jensenii*, *L. fermentum* NANDAN, and *L. crispatus* exhibited colonies with a white appearance, indicative of biofilm matrix inhibition (reduced curli and cellulose production—key constituents of biofilm matrix). These effects were comparable to those observed with the *E. coli* mutant strain MB4100BA, characterized by the absence of Curli subunits A and B.

**Figure 2:**
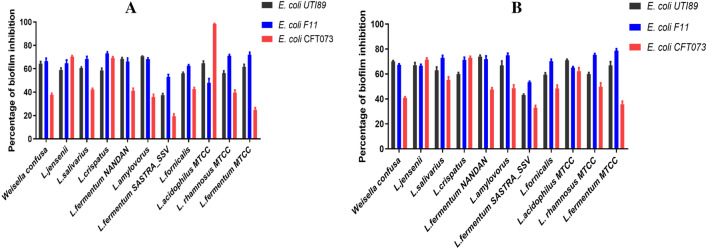
Percentage of Biofilm inhibition by Cell free Supernatants. (**a**) Bar graph showing the inhibitory effect of culture supernatants from isolated *Lactobacilli* strains on preformed biofilms of urinary tract infection (UTI) pathogens. The biofilm inhibition is expressed as a percentage relative to the control (no treatment). (**b**) Bar graph illustrating the inhibitory effect of culture supernatants from isolated *Lactobacill*i strains on biofilm formation of UTI pathogens. The biofilm formation inhibition is expressed as a percentage relative to the control (no treatment). Error bars represent standard deviation from the mean.

To evaluate whether *Lactobacilli* metabolites can curtail biofilm formation and/or disrupt preformed biofilms, cell free culture supernatant of *Lactobacilli* were either added initially or after 24 h post the formation of biofilms by UTI pathogens. The culture supernatants of all the isolated *Lactobacilli* caused ≥ 60% inhibition of both early and preformed biofilms of UTI pathogens (Fig. 2a, b). Specifically, among the UTI pathogens, *K. pneumonia, MDR K. oxytoca and C. koserii* exhibited higher susceptibility to the metabolites produced by the isolated *Lactobacilli* strains (Fig. S2), while the remaining *E.coli* UTI pathogens showed inhibition of both preformed biofilms and early biofilm formation. These findings underscore the significant anti-biofilm potential of human *Lactobacilli* isolates conferring colonization resistance against pathogens. Based on the antibiofilm potential, 3 strains (*L. fermentum NANDAN*, *L. crispatus*, and *L. jensenii*) that displayed potent antibiofilm activity were chosen for further studies.

### *Lactobacillus* CFS negatively impacts exopolysaccharide production and biofilm formation in uropathogenic *E. coli* UTI89

Biofilms are microbial communities encased within a self-produced extracellular polymeric substance (EPS) matrix, which plays a crucial role in their lifestyle. The matrix provides structural integrity, protection, and enables biofilms to exhibit various advantageous traits, including resilience to stress, social interactions, and architectural organization. we investigated the effect of CFS of *Lactobacilli* on the formation of biofilm matrix in UPEC strain UTI89. Our observations showed that the presence of *Lactobacillus* CFS significantly reduced the formation of *E. coli* UTI89 biofilms, compared to untreated control at 24 and 48 h respectively (Fig. S8).

### Scanning electron microscopy (SEM) imaging of *UPEC* biofilm inhibition by CFS

**Figure 3:**
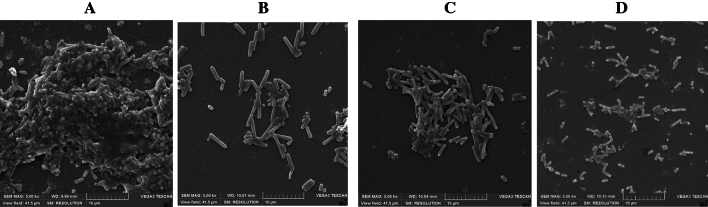
SEM images showing the morphological changes in *E. coli UTI89* biofilm treated with cell-free supernatant (CFS) derived from select *Lactobacillus* strains. (**A**) Untreated *E. coli UTI89* biofilm (**B**) *E. coli UTI89* biofilm treated with *L. jensenii* CFS (**C**) *E. coli UTI89* biofilm treated with *L. crispatus* CFS (**D**) *E. coli UTI89* biofilm treated with *L. fermentum NANDAN* CFS.

To assess the morphological changes induced by the cell-free supernatant (CFS) derived from selected *Lactobacilli* strains on biofilms of *E. coli* UTI89, scanning electron microscopy (SEM) analysis was performed*.* In the untreated control group, the biofilm exhibited complex and clumped structures characterized by the presence of matrix and multiple layers. The biofilm matrix appeared dense and compact, with interconnected network formations. *E. coli UTI89* cells were tightly adhered to each other and to the biofilm surface, indicating strong attachment and colonization (Fig. 3A). Conversely, the SEM analysis of the CFS-treated young biofilms revealed noticeable alterations in biofilm morphology. Compared to the untreated group, the CFS-treated biofilm displayed a reduced level of attachment. Treated samples displayed lack of sticky matrix with more dispersed and loosely organized structures, fewer clumps and thinner layers. *E. coli UTI89* cells exhibited reduced adhesion to the biofilm surface, indicating a decrease in cell attachment and colonization especially for the biofilms treated with CFS of *L. jensenii* and *L. fermentum* (Fig. 3B, C, D).

### Live/dead staining shows CFS induces cell death and inhibits biofilm formation in UPEC

Live dead staining of UTI89 cells exposed to CFS showed that CFS from *L. jensenii and L. fermentum* NANDAN showed a significant increase in proportion of dead cells implying that cell viability is drastically reduced upon treatment with CFS, which reinforces bactericidal potential of CFS against UPEC (Fig. S4). As *Lactobacilli* are well known to produce lantibiotics that kill the cells by affecting membrane viability, it is likely that antibacterial effect of UTI89 could also be mediated metabolites in CFS.

### Identification of tryptamine from CFS as the inhibitor of biofilm matrix

**Figure 4:**
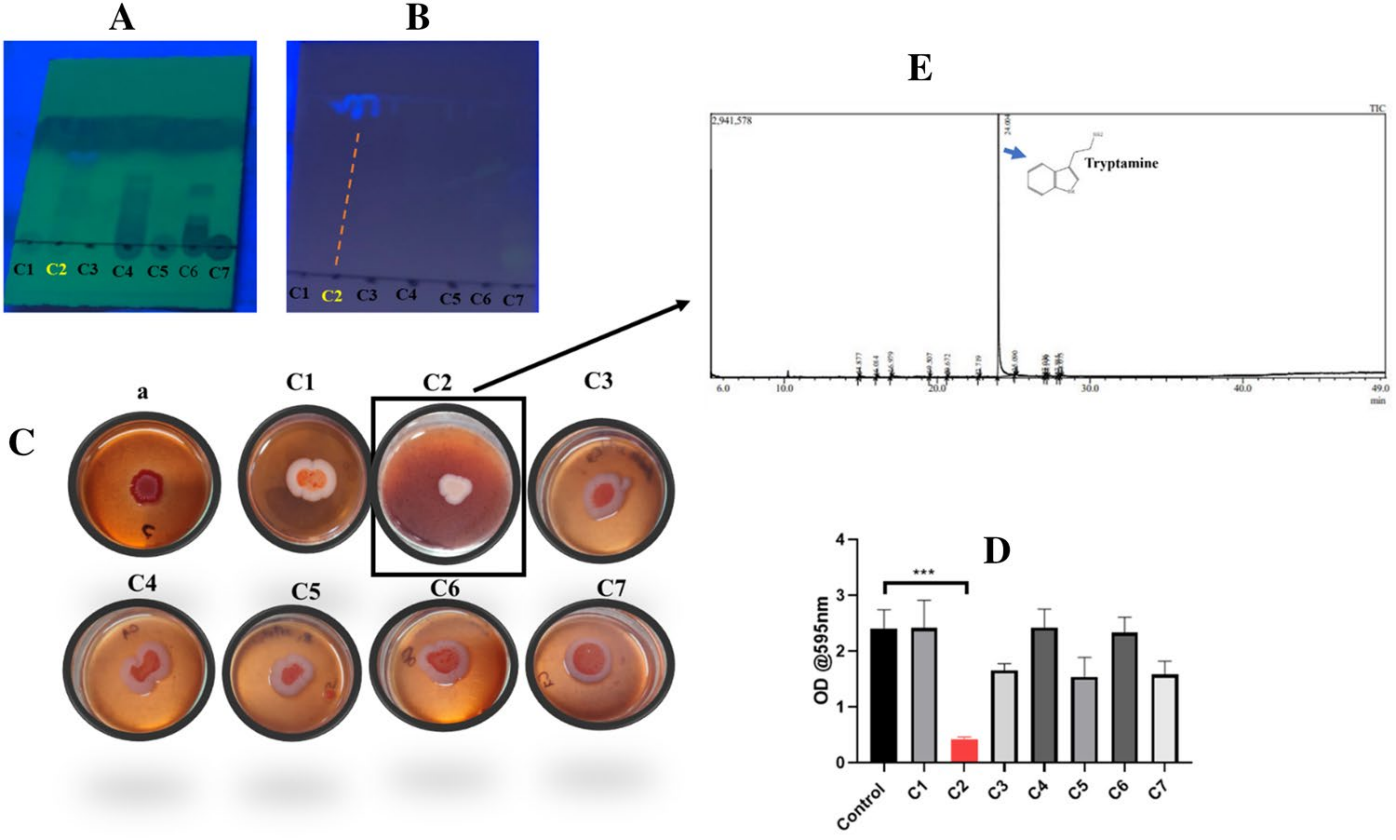
Screening and Characterization of Cell free supernatant from *Lactobacillus *sp. C1–C7 are Compounds 1 to Compound 7 (**A**) visualization of column chromatography purified fractions of CFS in TLC (**B**) TLC plate after UV exposure where C2 shows fluorescence marked by dotted yellow line (**C**) Screening of the C1 to C7 fractions for matrix inhibition, treatment with C2 fraction (marked in square) causes biofilm matrix inhibition, *E. coli* UTI89 colony biofilm phenotype was analyzed by inoculating it as a spot containing the metabolites on congo red media. Red, Dry and Rough (RDAR) colony morphology indicate matrix production, whereas Smooth and White (SAW) colony morphology indicate absence of matrix production (**D**) Crystal Violet assay showing ability of fractions to inhibit biofilm formation, mean ± SD from 3 replicates plotted for all panels. (**E**) GC–MS of C2.

We had separated seven fractions (compounds) from the Cell-free supernatant of *Lactobacillus* strains sourced from the human vaginal microflora (Fig. 4A). Among these Compound 2 fraction (C2) displayed fluorescence upon excitation with ultraviolet (UV) light (Fig. 4B). To evaluate the potential of these fractions (C1–C7) as matrix inhibitors, we employed the Congo Red assay, where the presence of Red, Dry, and Rough (RDAR) colony morphology indicated matrix production, while Smooth and White (SAW) colony morphology signified the absence of matrix. Notably, Compound 2 (C2) displayed significant matrix inhibition capabilities (Fig. 4C). Crystal violet assay for the fractions, demonstrated substantial reduction in the biomass of *E. coli* UTI89 biofilms following exposure to Compound 2 (C2) (Fig. 4D). To identify the specific chemical nature of this compound, we utilized gas chromatography-mass spectrometry (GC–MS) spectroscopy (Fig. 4E). Furthermore, our analysis using GC–MS revealed a prominent peak with a maximal area of 11,148,356 units and a peak height of 2,941,578 units. This peak manifested at a retention time of 24.004 min and exhibited a 95% similarity match to Tryptamine, a biogenic amine spectrum in NIST database, thus it is likely that the biofilm matrix inhibition mediated by C2 fraction could be mediated by Tryptamine. (Fig. S5; Table. S4).

### Validation of tryptamine as the anti-biofilm metabolite from CFS

**Figure 5:**
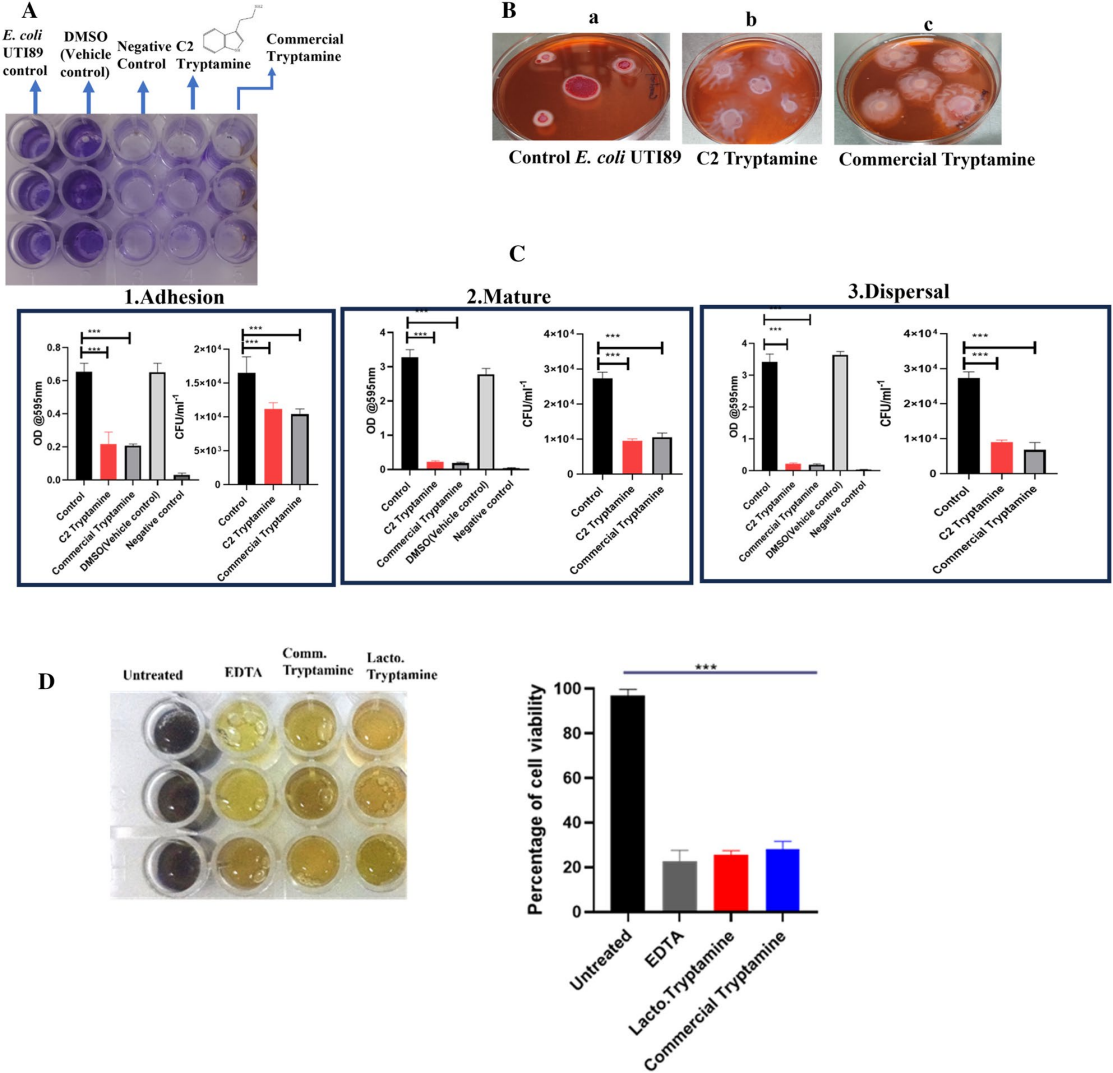
Inhibition of Matrix and biofilm formation by Tryptamine. (**A**) Crystal Violet Assay, 0,5% of DMSO which was used as a solvent to dissolve tryptamine acted as vehicle negative control (**B**) Matrix inhibition, where (**a**) Control *E. coli* UTI89 shows Red, Dry and Rough (RDAR) colony morphology indicate matrix production (**b**) and (**c**) C2 Tryptamine and Commercial tryptamine present in CFS Smooth and White (SAW) colony morphology indicate absence of matrix (**C**) The influence of tryptamine on biofilm development stages; (1) Adhesion (2) Mature (3) Dispersal n = 3,Statistical significance were analysed using one-way ANOVA***p < 0.001 (**D**) MTT assay showing Percentage Viability of preformed biofilm of *E. coli* UTI89 following treatment with *Lactobacillus* extracted tryptamine (Lacto. Tryptamine) and commercial tryptamine compared to untreated and positive control (EDTA-0.1%) conditions. Error bars represent the standard deviation from the mean (n = 3).

To validate if tryptamine causes inhibition of biofilm matrix in UPEC, commercially available tryptamine was procured from TCI chemicals (Tokyo Chemical Industry (India) Pvt Ltd., Chennai) and its ability to inhibit UPEC biofilms was evaluated relative to both CFS and C2 purified fraction. The minimum biofilm inhibition concentration (MBIC) was the lowest concentration preventing biofilm formation and minimum biofilm eradication concentration (MBEC) was the lowest concentration eradicating pre-formed biofilms both were determined using a microdilution assay. The MBIC for tryptamine was 4 µg/ml, while the MBEC for tryptamine was determined as 8 µg/ml (Fig. S6) further studies to test the effect of tryptamine at its MBIC/MBEC on distinct stages of biofilm development (Fig. 5A) was carried out.

*E. coli UTI89* colony morphology was analyzed by inoculating it as a spot CR media incorporated with 8 µg/ml of tryptamine. The presence of Red, Dry, and Rough (RDAR) colony characteristics signifies the production of an extracellular matrix (Fig. 5B), while the Smooth and White (SAW) colony attributes indicate the lack of extracellular matrix production. (Fig. 5Bb, Bc). The adhesion of *E. coli* UTI89 was reduced by > 50% (Fig. 5C.1) whereas biofilm formation was reduced by > 90% (Fig. 5C.2) and > 80% of biofilm eradication was observed due to tryptamine treatment (Fig. 5C.3). Our findings indicate a significant reduction in metabolic viability of the biofilm cells upon treatment with tryptamine and commercial tryptamine (8 μg/ml). The results were comparable to the effects observed with the positive control EDTA (0.1%), demonstrating that tryptamine effectively reduces the metabolic viability of biofilm cells (Fig. 5D). These results confirm that tryptamine not only inhibits biofilm formation but also disrupts the metabolic viability of established biofilms, further validating its antibiofilm potential. In order to test if tryptamine could inhibit curli production, which could have potentially disrupted the adhesion between the bacterial cells and abiotic surfaces, we quantified the level of curli production following tryptamine treatment by performing congored (CR) binding assay, wherein around fourfold reduction in CR binding to the *E. coli* UTI89 was observed implying reduced curli production due to tryptamine treatment (Fig. S7).

### *Lactobacillus* CFS/tryptamine exerts bactericidal effect against biofilm derived cells of UPEC

**Figure 6:**
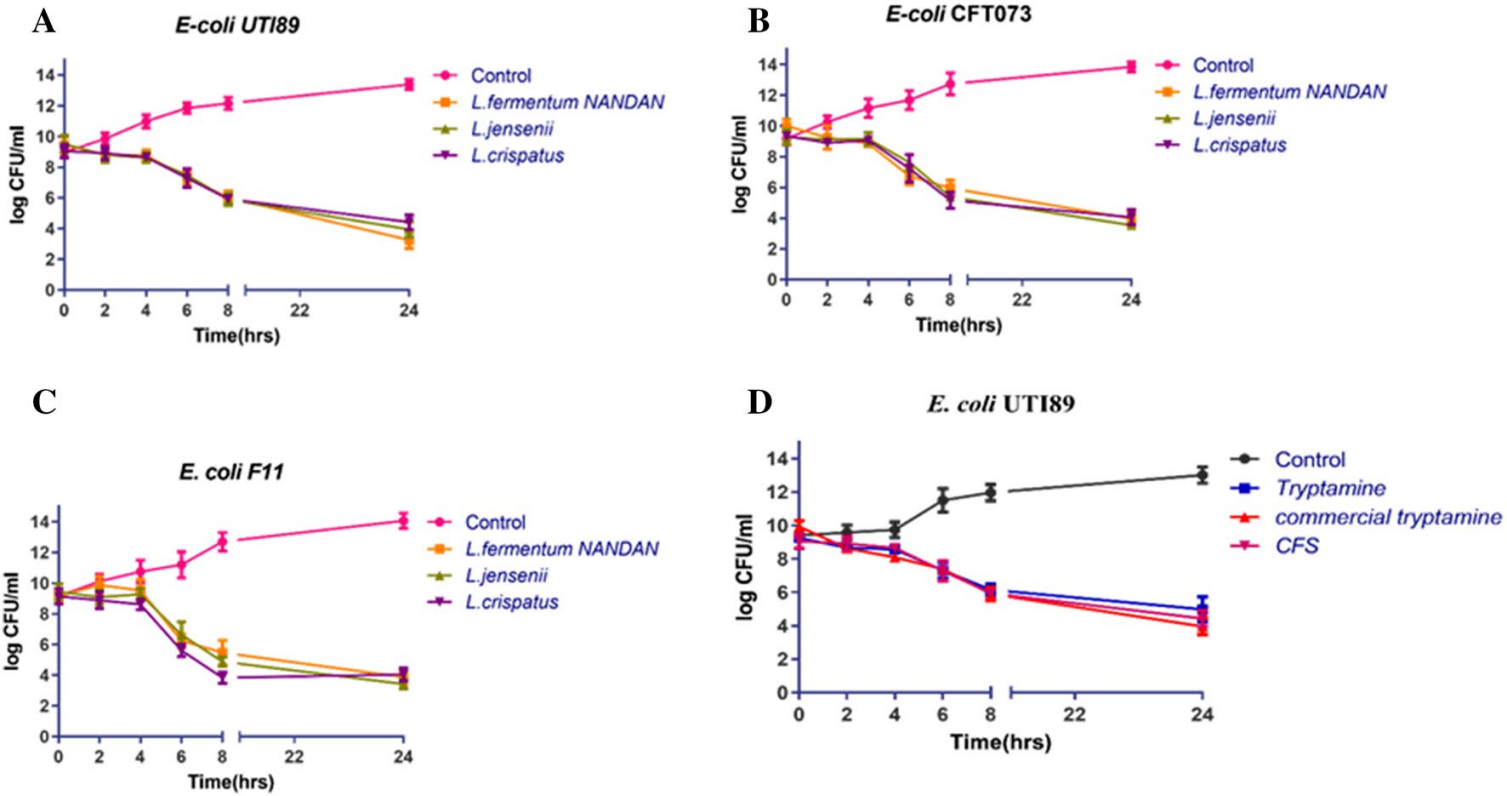
Time-kill assay evaluating the efficacy of cell-free supernatants from three selected *Lactobacilli* strains (*L. fermentum NANDAN, L. crispatus, and L. jensenii*) against biofilm derived cells of uropathogens. Each panel in the figure represents the effect of the *Lactobacilli* strains on a specific uropathogen. (**a**) Effect on *E. coli UTI89* (**b**) Effect on *E. coli CFT073* (**c**) Effect on *E. coli* F11 (**d**) Effect of tryptamine and commercial tryptamine on *E. coli* UTI89, with untreated and CFS treatments as negative and positive controls respectively. The error bars represent the standard deviation among the three replicates.

Time-kill assays were conducted on preformed biofilms, exposing them to cell-free supernatants (CFS) from *Lactobacillus fermentum* NANDAN, *Lactobacillus crispatus*, and *Lactobacillus jensenii*, evaluating the efficacy of both CFS and tryptamine isolated from *Lactobacillus* against UPEC viz., *E. coli* UTI89 (Fig. 6a), *E. coli* CFT073 (Fig. 6b) and *E. coli* F11 (Fig. 6c). Notably, all Lactobacillus CFS treatments resulted in a remarkable four-log reduction. Additionally, experiments were conducted with both isolated tryptamine from CFS, and commercially procured tryptamine, specifically with *E. coli* UTI89 as a representative pathogen, and results were comparable for both tryptamines (Fig. 6d). Furthermore, experiments were carried out with CFS on clinical strains of *Klebsiella* spp. (MDR *K. oxytoca* and *K. pneumoniae*) (Fig. S9). Both *L. jensenii* and *L. crispatus* demonstrated similar effects against the biofilm-derived cells of uropathogens, suggesting that both CFS and tryptamine exert a bactericidal effect on biofilm cells of uropathogens.

#### Bactericidal effect of CFS and tryptamine is mediated by ROS

**Figure 7:**
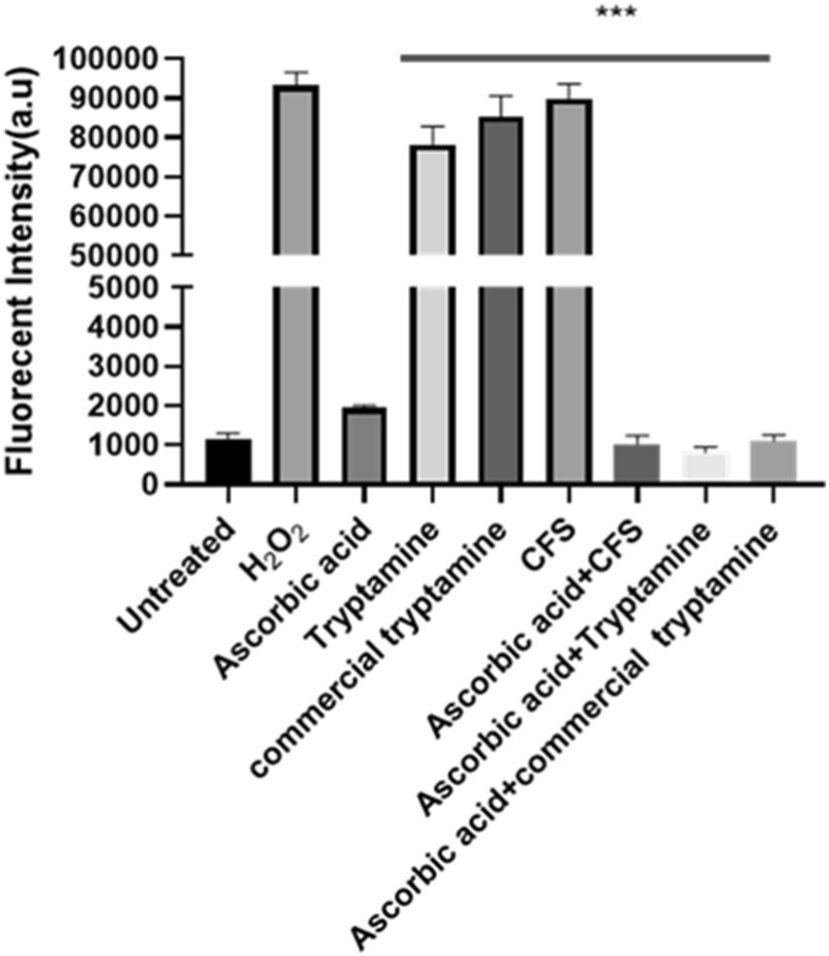
Comparative analysis of reactive oxygen species (ROS) generation in uropathogenic *E. coli* UTI89 biofilms treated with Tryptamine and cell-free supernatant (CFS). The experimental group treated with *Lactobacillus* CFS, and tryptamine exhibited a significant increase in ROS generation compared to the control group. Statistical significance was determined using Student’s t-test (***p < 0.001).

As the CFS exhibited bactericidal effect against biofilm derived cells of uropathogens, we were curious to know if the bactericidal effect was mediated by generation of reactive oxygen species (ROS). Hence ROS assay was performed on cells exposed and unexposed to CFS and H_2_O_2_ was used as the positive control. We also used ascorbic acid (100 mM), a well-known ROS inhibitor, to determine whether the ROS generation observed is indeed attributable to the cell-free supernatant (CFS) and tryptamine. The uropathogenic biofilms treated with cell-free supernatant (CFS) derived from chosen *Lactobacillus* strains, exhibited a statistically significant elevation in ROS generation relative to the untreated group. Addition of ascorbic acid along with CFS/tryptamine significantly reduced ROS levels implying that bactericidal effect of CFS/tryptamine is actually mediated by the ROS (Fig. 7).

In fact, the amount of ROS produced by treatment with *L. fermentum* CFS was comparable to ROS generated by H_2_O_2_ (Fig: S10). Similarly, tryptamine treatment on *E. coli* UTI89 showed a comparable increase in ROS production, indicating its antibacterial potential like CFS (Fig. 7). These findings suggest that both CFS and tryptamine possess significant antibacterial properties, which could be promising in combating uropathogenic biofilms and could potentially contribute to biofilm disruption.

Probiotic strains also aid in protecting host tissue against oxidative damage. Towards this end, radical scavenging assay was performed for CFS of *Lactobacilli* strains. Relative to the control strains, *L. rhamnosus* MTCC, *L. fermentum* MTCC and *L. acidophilus* MTCC (95.03 ± 1.20, 86.23 ± 0.7 and 90.4 ± 0.9 respectively), the Cell-free supernatant (CFS) of the isolated *Lactobacilli* strains displayed high DPPH scavenging activity. With *L. fornicalis* (98.15 ± 0.7) and *L. fermentum* (95.22 ± 1.5) strain SASTRA SSV displaying the highest radical scavenging activity and *L. jensenii* displaying the least amount of activity (66.83 ± 1.53). We found it interesting that different strains of *L. fermentum* displayed varied radical scavenging activities. (75.19 ± 1.46 and 95.22 ± 1.5). (Fig. S11).

#### CFS but not tryptamine, impairs motility and cell adhesion in UPEC biofilms

To gain mechanistic insights into the antibiofilm (inhibition of biofilm formation) effect, motility and cell adhesion assays were undertaken. The results demonstrated a significant impact of CFS treatment on both swarming and swimming motility of *E. coli* UTI89. Notably, the swarming and swimming motility of *E. coli* UTI89 were impaired in the presence of CFS at both the MIC and sub-MIC concentrations. The inhibition of swarming motility was most pronounced at the MIC level, wherein the migration distance of *E. coli* UTI89 was notably reduced relative to control conditions (without CFS). (Fig. S12, Table. S5). Conversely, experiments with tryptamine, both isolated and commercially procured, revealed no inhibition of swimming and swarming motility at both MIC and sub-MIC levels (Fig. S13, Table S6). This implies that additional unexplored metabolites in CFS targets flagellar and pili-based motility.

#### CFS/tryptamine alters gene expression in biofilm derived cells of *E. coli* UTI89

**Figure 8:**
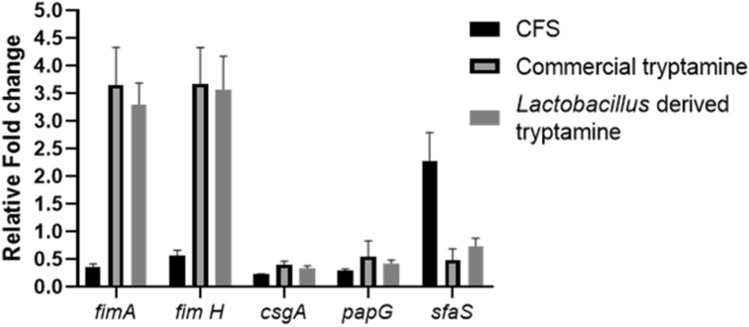
Effect of Cell Free Supernatant of *L. jensenii* on the expression of biofilm genes in *E-coli* UTI89. After normalisation with the housekeeping gene 16srRNA, the gene expression levels were evaluated using ∆∆ct method and expressed as a relative fold-difference in Gene expression. Mean ± SD from 3 replicates plotted for all panels.

For planktonic UPEC (uropathogenic *Escherichia coli*) to transition into a sessile biofilm state, a precise and coordinated regulation of critical genes associated with biofilm formation is required^45^. To understand the impact of CFS on gene expression of select genes, we performed qPCR on key UPEC genes (*fimA, fimH, papG, csgA, sfaS*) known to be associated with biofilm formation and virulence. The primers used are listed in Supplementary Table: S7. The initial adhesion process required for triggering biofilm formation in *E. coli* UTI89 is mediated by Type I pili^46,47^. Two major subunits of the Type I pili are FimH and FimA, encoded by the *fimH* and *fimA* genes, respectively^48,49^. Additional genes tested include S-fimbriae *(sfaS*) and P-fimbriae (*papG*), both of which are crucial virulence factors for inducing urinary tract infections (UTI) and promoting biofilm formation^50,51^ and *csgA,* which contributes to the production of curli in *E. coli*. When UPEC was treated with CFS, we observed a significant reduction in the expression of *fimH* and *fimA* genes compared to the control. Furthermore, CFS treatment also led to a substantial reduction in the expression of *papG* and curli (csgA) genes. Among the genes evaluated, only S-fimbriae adhesion (*sfaS*) showed increased gene expression during CFS treatment (Fig. 8). This suggests that the antibiofilm effect of CFS could be attributed to impairment in initial adhesion process and reduced matrix forming ability. On the other hand, tryptamine treatment appears to have a contrasting effect on biofilm formation. It increases the expression of *fimA* and *fimH* genes and reduces the expression *csgA*, *papG* and *sfaS*, which implies that by preventing adhesion and curli production, tryptamine could prevent surface colonization and thereby exert its antibiofilm effect. Additionally, the expression of the *sfaS* gene was reduced, which is contrary to the findings with CFS. Thus, the downregulation of genes involved in adhesion and curli production mediated by tryptamine, culminates as inhibition in biofilm formation.

#### Tryptamine induces depletion of biofilm matrix components

The assessment of matrix formation was conducted using a Congo red depletion assay, where an increase in absorbance in the supernatant indicates a decrease in matrix constituents. The minimal matrix production was observed following treatment with 8 µg/ml (MBEC concentration) of tryptamine (Fig: S14).

#### Tryptamine does not affect cellulose content in biofilms

Calcofluor exhibits affinity for cellulose, and the quantification of bound Calcofluor through spectrofluorimetry inversely correlates with the quantity of cellulose produced by various biofilms. Our observations indicate that the presence of tryptamine does not significantly alter the cellulose content (Fig. S15). This suggests that the primary target of tryptamine action is curli component of the biofilm matrix.

To determine the impact of tryptamine on the source strain *Lactobacillus,* we performed experiments using tryptamine at a concentration of 8 µg/ml, demonstrating that tryptamine at this concentration did not inhibit the growth or biofilm formation of *Lactobacillus* strains, as evidenced by the lack of significant differences in biofilm formation in both initial (Fig S18A) and preformed (Fig. S18B) *Lactobacillus* biofilms.

Thus, we were able to show that CFS from *Lactobacilli* caused significant inhibition in biofilm formation by UPEC which is primarily attributed to the ability CFS to induce ROS. Purification and Characterization of metabolites from CFS revealed that tryptamine present in CFS inhibits both biofilm formation and biofilm dispersion, which is mediated by reduction in formation of curli, but not cellulose. Gene expression profiling reveals that reduced expression of *fim*A, *fim*H and *Pap*G might be attributed to inhibitory effect on biofilm formation whereas decreased production of curli could be responsible for weak biofilms resulting in easier eradication of preformed biofilms.

## Discussion

A biofilm matrix typically consists of proteins, polysaccharides, and DNA. The release of the cells trapped in the matrix and the subsequent spread of the infection are both facilitated by the pathogen-specific enzymes that break down these matrix components^52^. The purpose of the present study was to identify the diversity and antibiofilm potential of culturable *Lactobacilli* from a small south Indian cohort, to characterise them at the molecular level, and to assess the key characteristics needed to create an effective vaginal probiotic in an era where antimicrobials continue to fail. The vaginal *Lactobacillus* species predominantly inhabit the vaginal and periurethral regions, serving as commensal organisms crucial for impeding pathogen adherence and migration to the bladder urothelium^53^. Decrease in vaginal *Lactobacilli* levels has been demonstrated to correlate with a higher incidence of vaginal colonization by *E. coli*^54^. These microorganisms engage in competition with uropathogens for adhesion to the receptors on the vaginal epithelium, effectively hindering the colonization by uropathogenic organisms^55^. The process of adhesion can be inhibited through various mechanisms, including exclusion, competition, and displacement. Exclusion involves occupying binding sites to prevent initial attachment by uropathogens. Competition entails directly vying with uropathogens for adhesion receptors on vaginal epithelial cells. Displacement involves removing uropathogens that have already bound to vaginal epithelial cells^56–58^.

The present work highlights the antibiofilm potential of metabolites from the cell free supernatant of *Lactobacillus*, which inhibits biofilm formed by uropathogens. In the present study, the isolated strains were deemed safe to use as probiotics as they lacked haemolytic activity and fibrinogen adhesion ability (Fig. S16 & Fig. S17). All the Lactobacilli isolates employed in this study exhibited auto aggregation characteristics (Table S8) the degree of auto aggregation appeared to depend on the strain. Bacterial colonisation and adhesion are likely affected by auto-aggregation. As it promotes colonisation through the formation of a bacterial film and aids in the exclusion of pathogens, evaluating auto-aggregative potential is a sign of adhesion property. Apart from *L. fermentum* strains, all isolates exhibited strong adhesion to xylene (Table S9), a non-polar solvent, demonstrating enhanced cell surface hydrophobicity of these isolates, which in turn disfavours pathogen adherence. Previous reports showed that the relative abundance of glycoprotein on the surface of microorganisms, contributed to greater levels of hydrophobicity, whereas the presence of polysaccharides was linked to hydrophilic surfaces^59,60^. From our coaggregation studies, we found out that the isolates could coaggregate with the clinically relevant uropathogens such as MDR *Klebsiella oxytoca*, *Klebsiella pneumoniae*, *Citrobacter koserii*, Uropathogenic *E. coli* UTI89 and *E. coli* CFTO73 strains (Table S10) and thus have the potential to prevent pathogens from adhering on to mucosal surfaces thereby affording colonization resistance. The coaggregation of lactobacilli with uropathogens leads to the formation of a microenvironment where antimicrobial substances produced by lactobacilli, such as hydrogen peroxide, lactic acid, and bacteriocin, are concentrated near the uropathogens. This concentration effectively inhibits bacterial biofilm formation and downregulates the production of pro-inflammatory cytokines such as tumor necrosis factor, IL-6, IL-8, and IL-10^61,62^. Another in vitro study evaluating 15 distinct *Lactobacillus* strains showed that *Lactobacillus crispatus* exhibited superior efficacy in impeding uropathogen adherence to vaginal epithelial cells compared to other lactobacilli examined, while certain strains such as *Lactobacillus jensenii* demonstrated heightened capability by directly restraining the growth of uropathogens^57^.

*Lactobacilli* employ several mechanisms to inhibit vaginal and urethral colonization by uropathogens, including competing for nutrients, generating substances directly toxic to uropathogenic bacteria (e.g., hydrogen peroxide, bacteriocins, and bacteriocin-like compounds), and inducing an acidic vaginal pH through lactic acid production^53,63^. Specific *Lactobacillus* strains, namely *L. salivarius* UCM572 and *L. acidophilus* 01, were earlier reported to hinder the adhesion of typical uropathogens to the bladder urothelium, suggesting a broader role beyond just vaginal colonization^64^. An earlier report showed that the organic acids secreted by *Lactobacillus* spp. isolated from urine, was effective against uropathogenic *Proteus mirabilis*^65^.

Bioassay guided screening and characterization of the cell free supernatant of *Lactobacillus* by and GC–MS, led to the identification of tryptamine, (Fig. 4, Fig. S5, Table. S3) which mediated inhibitory effects on the biofilm matrix of *E. coli* UTI89. Tryptamine, a monoamine derived from tryptophan and containing the indole structure, is found in significant quantities in the faeces of both humans and rodents^66^. It was shown that bacterial metabolism of tryptophan generates tryptamine, while the metabolic pathway is not prevalent among most human commensal bacteria, recent discoveries have identified the expression of tryptophan decarboxylase, the enzyme responsible for converting tryptophan to tryptamine, in *Ruminococcus gnavus* and *Clostridium sporogenes*^67,68^. Literature suggests that specific strains of *Lactobacillus bulgaricus* and *L. curvatus* exhibit the capacity to synthesize tryptamine^69,70^. Indole alkaloids represent a substantial group of naturally occurring compounds known for their diverse biological properties. These compounds have been found to exhibit a wide range of effects including anticancer, antimalarial, hallucinogenic effects^71–73^.

Tryptamine works in the context of the host epithelial cell lining by activating the 5-HT4 receptor, which is ubiquitously expressed along the colonic epithelium^74^. 5-HT4 receptors are also present in the vagina, cervix and endometrium, which are parts of the female reproductive system^75^. Tryptamine binds to the 5-HT4 receptor on the epithelial cells, causing a conformational change in the receptor. This binding activates the Gs alpha subunit, which exchanges GDP for GTP and liberates the Gs alpha subunit from the 5-HT4 receptor and βγ subunit. The GTP-bound Gs alpha subunit activates adenylyl cyclase, which in turn catalyzes the conversion of ATP into cyclic adenosine monophosphate (cAMP). Activation of these receptors can influence cell signaling pathways that regulate inflammation, cell proliferation, and local immune responses^74^. Tryptamine is an agonist for Trace Amine-Associated Receptors (TAARs), which are G-protein coupled receptors (GPCRs) that are activated by trace amines. TAARs are involved in various physiological processes, including immune responses, neurotransmission, and sensory perception. Activation of TAARs in the vaginal epithelium could modulate local immune responses, which are critical for maintaining reproductive health and preventing infections. TAARs can influence microbial populations in the vaginal epithelium by regulating the activity of bacteria and other microorganisms^76,77^. Tryptamine is also the substrate for Monoamine oxidase (MAO), MAO-A protein located in the cytoplasm of the vaginal epithelial cells. The metabolic by-products of tryptamine oxidation by MAO enzymes can contribute to oxidative stress in the vaginal epithelium^78,79^. The impact of tryptamine on cell junctions and epithelial integrity can affect the mucosal barrier, influencing the susceptibility to infections and the maintenance of a healthy environment. Tryptamine can also affect adherens junctions (AJs), which are involved in the formation of the apical junctional complex (AJC) and play a role in maintaining epithelial barrier integrity. Tryptamine can improve epithelial barrier function by increasing the expression of Tight junction (TJ) proteins, such as ZO1 and occludin, and apical junctional proteins, such as E-cadherin and β-catenin^80^.

Tryptamine specifically affected all the three major stages of biofilm formation viz., adhesion, maturation, and dispersion (Fig. 5C) implying that tryptamine not only inhibits biofilm formation it can also effectively disrupt mature biofilms. Congo red depletion and calcofluor staining assay (Fig. S14, Fig. S15) specifically showed that tryptamine affects curli production but not cellulose production. As curli contributes structural rigidity of biofilm matrix^81^, reduction in curli results in weak or poor biofilms. In fact, previous study showed that the curli deficient *E. coli* cells fail to form mature 3D biofilms and results in the formation of single layer of cells^82^. Moreover, curli is an ideal pathogenic molecular signature that is recognised by the host immune system through TLR1/TLR2 receptors resulting in production of IL-6, IL-8, TNF alpha and nitric oxide^83^. As biofilms are difficult to eradicate, continued presence of curli from biofilms, could result in aggravated immune response leading to persistent inflammation that potentially damages host^82^. In fact an earlier study had shown that *Lactobacilli* co aggregating with uropathogens inhibits biofilm formation by uropathogens and downregulates the production of pro-inflammatory cytokines such as TNF alpha, IL-6, IL-8, and IL-10^61^. Thus tryptamine by indirectly reducing curli, can possibly modulate the immune response positively.

Time kill studies on biofilm derived cells (Fig. 6, S9), fluorescent live-dead staining (Fig S4) have shown that the CFS/tryptamine has a strong bactericidal effect and significantly impacts the viability of the *E. coli* UTI89 cells. It is noteworthy that tryptamine exhibits toxicity towards bacteria, yeast, and plants. Concentrations of tryptamine less than or equal to 20 μg/mL impede the growth of most cyanobacteria and eukaryotic microalgae^84^. Previous studies have shown that Tryptamine, when present at concentrations lower than Tryptophan (Trp), induces a deficiency in tryptophanyl-tRNA, resulting in a subsequent decrease in protein translation within gut bacteria. Furthermore, tryptamine exhibits a high affinity for the surfaces of pathogenic microorganisms such as *E. coli*, *Candida albicans*, and *Pseudomonas aeruginosa*^85^. Tryptamine’s cytotoxic and inhibitory effects on cellular processes, may contribute to its bactericidal effect.

To aid in pathogen mitigation, ROS is a potent tool as ROS is known to exhibit significant microbicidal effect^86^. The ability of CFS and tryptamine from *Lactobacilli* to induce ROS comparable to that generated by H_2_O_2_ (Fig. 7, Fig S10), imply that probiotic *Lactobacilli*, isolated from healthy human vagina, probably exerts its antimicrobial activity on biofilm derived cells through tryptamine mediated ROS. Previous study has demonstrated that the toxic metabolites produced by lactobacilli, including hydrogen peroxide and lactic acid, act cooperatively to effectively eradicate uropathogenic organisms in vitro, with hydrogen peroxide showing increased killing potency in the presence of lactic acid^87^.

Differential gene expression with Cell free supernatant (CFS) showed downregulation of genes related to Type 1 pili, P type fimbriae and curli. The reduced cell surface hydrophobicity most likely contributed by decreased expression of Type 1 Pili, P type fimbriae, in addition to reduction in curli (matrix) formation is likely to result in significantly impaired biofilm formation by CFS treated cells relative to untreated cells. Interestingly, CFS but not tryptamine displayed the ability to inhibit swimming and swarming motility (Fig. S12, Table S5 and Fig. S13, Table S6) which might also hinder early stages of biofilm formation or subsequent maturation. Although tryptamine treatment induced the upregulation of *fimA* and *fimH* genes associated with adhesion, it also led to the downregulation of P-type fimbriae (*PapG*) s-fimbriae (*sfaS*) and curli (*csgA*) genes, suggesting a targeted disruption of biofilm-associated adhesion factors without affecting motility. The contrasting effects of CFS and Tryptamine on Type 1 pili gene expression and motility underscore their distinct modes of action in inhibiting biofilm formation (Fig. 8, Fig. S13). While CFS exerts a broad inhibitory effect on gene expression and motility, Tryptamine appears to selectively modulate adhesion-related gene expression without impacting motility. It is likely that Tryptamine’s high affinity for the surfaces of pathogenic microorganisms, including *E. coli*, and its ability to downregulate genes involved in P type and S type fimbriae (Fig. 8) may lead to direct disruption of the pathogens ability to adhere to surfaces and form biofilms. This could be a key factor in its biofilm inhibition properties, as it prevents the initial stages of biofilm formation (Fig. 5C). The overall antibiofilm activity of the CFS appears to involve multiple mechanisms, including the downregulation of key biofilm-associated genes such as *fimA, fimH, papG,* and *csgA*. The increase in *sfaS* expression, while intriguing, may reflect a compensatory response or an alternative pathway that the bacteria engage in, when primary adhesion mechanisms are inhibited. The expression of S-fimbriae can be phase variable, allowing the bacteria to switch between different adhesion mechanisms to adapt to changing environments^88^. Biofilm formation is a highly complex process regulated by numerous genetic and environmental factors. The increased expression of *sfaS* could be an attempt by the bacteria to compensate for the inhibited functions of other adhesion structures (Type I pili and P-fimbriae). In the presence of shear forces, the S-fimbriae adhesion can enhance its binding capacity through catch-bond mechanisms, providing a selective advantage in turbulent conditions. V77A and L79P mutations in *sfaS* gene can lead to a strong increase in initial adhesion, but have little impact on biofilm formation capacity^89^. Despite this upregulation, the dominant effect of CFS on other critical genes involved in adhesion and matrix formation could still lead to an overall reduction in biofilm formation. The expression of S-fimbriae might be influenced by the presence of other adhesins. In some cases, the coexistence of different adhesins can lead to a trade-off between specific and nonspecific adhesion properties, affecting biofilm formation^90^. The S-fimbriae (*sfaS*) might play a role only in specific stages or under certain conditions of biofilm formation, it may not fully counteract the inhibitory effects on the primary adhesion mechanisms. For example, *sfaS* might be involved in secondary adhesion processes or in maintaining cell–cell interactions within the biofilm rather than initial surface attachment^91^. The post-transcriptional and post-translational modifications can decouple gene expression changes from functional outcomes^92^. The actual functionality of S-fimbriae proteins and their impact on biofilm formation may be limited even if their gene expression is upregulated. Hence the increase in *sfaS* gene expression might not undermine the overall antibiofilm efficacy of CFS, which is primarily mediated by the downregulation of other critical biofilm-associated genes. Furthermore, our assessments through the congo red assay and calcofluor binding assay also revealed significant reduction in curli production with no impact on cellulose (Fig: S7 & Fig. S15). By inhibiting curli production, tryptamine weakens the structural integrity of biofilms.

Thus CFS of *Lactobacilli* exerts antibacterial effect through ROS, inhibits swimming and swarming motility (Fig S12), and reduces the gene expression of Type 1 Pili related genes and curli (Fig. 8 ) resulting in impaired matrix production (Fig. 5 ), the bactericidal and matrix inhibitory effect with reduced curli formation is attributed to tryptamine present in CFS. Infact, tryptamine hinders all three stages of biofilm formation resulting in poor biofilms, which mechanistically underscores the antibiofilm potential of tryptamine. By impairing protein synthesis and affecting microbial cell viability, tryptamine contributes to bactericidal effect of biofilm cells. Some *Lactobacillus* species, such as *Lactobacillus plantarum*, *Lactobacillus acidophilus*, and *Lactobacillus rhamnosus*, have been found to produce tryptamine in vitro^74^. *Lactobacillus* strains can tolerate tryptamine concentrations up to 8 mM, which is higher than the levels produced by some gut bacteria in vitro^24^. In our experiments, we assessed the impact of tryptamine on *Lactobacillus* at a concentration of 8 µg/ml. The results showed that this concentration of tryptamine did not inhibit the growth or biofilm formation of *Lactobacillus* strains. Both initial and preformed biofilms of *Lactobacillus,* treated with tryptamine did not exhibit any significant differences in biofilms relative to the untreated control (Fig. S18A & S18B). Thus tryptamine, at the tested concentration, does not negatively impact the biofilm-formation or the stability of preformed biofilms of *Lactobacilli*, suggesting that it does not adversely affect the structural integrity of *Lactobacilli* biofilms. This finding is significant as it highlights that tryptamine can be used in therapeutic applications targeting UPEC biofilms without compromising the beneficial biofilms formed by *Lactobacillus.* To the best of our knowledge, there appears to be only one study documenting the inhibitory effects of tryptamine (indole derivatives) on *Candida albicans* biofilm formation^93^. Tryptamines antibiofilm effect against other microbes are hitherto unreported. Thus, our study has identified tryptamine as the potent antibiofilm agent from the supernatant of *Lactobacilli* that can effectively thwart biofilm formation by reduced adhesion and matrix production, and it exert bactericidal effect on biofilm cells by generating ROS thereby mitigating UTI. Efficacy of CFS/tryptamine to prevent colonization by UPEC in animal models will be evaluated in future studies.

## Conclusion

In conclusion, our study highlights the significant potential of probiotics, specifically *Lactobacillus*-derived tryptamine, as a novel prophylactic approach against biofilms, particularly those caused by Uropathogenic *Escherichia coli* (UPEC). We have isolated vaginal commensal microbiota from healthy Indian women and explored their probiotic traits, shedding light on their ability to inhibit UPEC biofilm formation. Our research revealed that the tryptamine from the cell-free supernatant (CFS) of *Lactobacilli* has a profound impact on UPEC. It reduced UPEC’s cell surface hydrophobicity, hindered matrix production by downregulating key genes associated with biofilm formation and exerted bactericidal effect on biofilm derived cells through ROS. Overall, our study offers promising insights into the potential therapeutic applications of probiotics/ tryptamine in combating UPEC biofilm formation. These findings provide a valuable foundation for future research in biofilm inhibition and microbial physiology, offering exciting prospects for novel strategies to combat biofilm-related infections.

### Supplementary Information

Supplementary Information.

## Data Availability

The 16srRNA sequence datasets generated and/or analysed during the current study are available in the NCBI GenBank repository, The organism name and the accession number are as follows: *Ligilactobacillus salivarius strain SSV* (Accession No: *OP642364*), *Lactobacillus crispatus strain SSV* (Accession No:*OP642371), Lactobacillus fornicalis strain SSV* (Accession No: *OP642370), Lactobacillus jensenii strain NANDAN* (Accession No: *OP648111), Limosilactobacillus fermentum strain NANDAN* (Accession No: *OP648129), Weissella confusa strain NANDAN* (Accession No: *OP648134), Limosilactobacillus fermentum strain SASTRA_SSV* (Accession No: *OP658868), Lactobacillus amylovorus strain SASTRA_SSV* (Accession No: *OP658873), Ralstonia insidiosa strain SSV* (Accession No: OP630602), *Ralstonia syzygii* subsp. indonesiensis strain SSV (Accession No: OP630599), *Enterococcus faecalis* strain SSV(Accession No: OP642363), *Pandoraea pnomenusa strain SASTRA-3* (Accession No: OP648138), *Ralstonia insidiosa strain MBL* (Accession No: OP649852), *Enterococcus faecalis strain SASTRA-2* (Accession No: OP648136). All pertinent data has been thoroughly detailed in the main text and supplementary materials. Raw data can be shared upon request to sai@scbt.sastra.edu.
