# Supplementary material for: Biogenic amine tryptamine in human vaginal probiotic isolates mediates matrix inhibition and thwarts uropathogenic E. coli biofilm
Source: Sci Rep. 2024 Jul 4;14:15387. doi: 10.1038/s41598-024-65780-0 (PMC11224256; doi:10.1038/s41598-024-65780-0)
Supplement: Supplementary file 1 — Supplementary Information. [file 41598_2024_65780_MOESM1_ESM.pdf]

SUPPLEMENTARY

Biogenic Amine Tryptamine in Human Vaginal Probiotic Isolates Mediates Matrix Inhibition and Thwarts Uropathogenic *E. coli* Biofilm.

Veena G Nair [1,6], C.S. Srinandan[1], Y.B.R.D Rajesh[2], Dhiviya Narbhavi[2], Anupriya A[3], Prabhusaran N[4,] Sai Subramanian N [6*]

* **Corresponding Author:** Dr Sai Subramanian N, Antimicrobial Resistance Lab, Centre for Research in Infectious diseases, School of Chemical and Biotechnology, SASTRA Deemed to be University, Thanjavur 613401, Tamil Nadu, India. Phone: +91 4362 2641011,

email: sai@scbt.sastra.edu

[1] Microbial Biofilm Lab, Centre for Research in Infectious diseases, School of Chemical and Biotechnology, SASTRA Deemed to be University, Thanjavur 613401, Tamil Nadu, India. Phone: +91 4362 264101

[2]Department of Chemistry, School of Chemical and Biotechnology, SASTRA Deemed University, Thanjavur – 613 401, Tamil Nadu, India.

[3] Assistant Professor, Department of Obstetrics and Gynaecology, TSRMMCH&RC, Tiruchirappalli, Tamil Nadu, India

[4]Associate Professor, Department of Microbiology, TSRMMCH&RC, Tiruchirappalli, Tamil Nadu, India

[5] Research Faculty, Institutional Research Board TSRMMCH&RC, Tiruchirappalli, Tamil Nadu, India

[6]Antimicrobial Resistance Lab, Centre for Research in Infectious diseases, School of Chemical and Biotechnology, SASTRA Deemed to be University, Thanjavur 613401, Tamil Nadu, India.

Demography	
Age (years ± SD)	33.4 ± 5.4
Weight (kg ± SD)	65.6 ± 10.9
Height (cm ± SD)	150.4 ± 6.4
BMI (kg/m^2)	27.8 ± 4.1
Literacy (%)	High school: 33%<br>Higher secondary: 3.7%<br>Diploma/College: 20%<br>Illiterate: 35%
Occupation (%)	Not working: 75%<br>Working: 20%
Diet (%)	Vegetarian: 9%<br>Mixed Diet: 89%
Blood Group (%)	A^{+ve}: 35%<br>AB^{+ve}: 18%<br>B+^{ve}:16%<br>O^{+ve}: 26%

Table. S1 Demographic details of participated Indian women in the collection of vaginal swabs.

Strain	*Accession No:*
Ligilactobacillus salivarius strain SSV	*OP642364*
Lactobacillus crispatus strain SSV	*OP642371*
Lactobacillus fornicalis strain SSV	*OP642370*
Lactobacillus jensenii strain NANDAN	*OP648111*
Limosilactobacillus fermentum strain NANDAN	*OP648129*
Weissella confusa strain NANDAN	*OP648134*
Limosilactobacillus fermentum strain SASTRA_SSV	*OP658868*
Lactobacillus amylovorus strain SASTRA_SSV	*OP658873*

Table. S2 GenBank accession numbers of isolated *Lactobacillus* sps

Strains	Accession Number
Ralstonia insidiosa strain SSV	OP630602
Ralstonia syzygii subsp. indonesiensis strain SSV	OP630599
Enterococcus faecalis strain SSV	OP642363
Pandoraea pnomenusa strain SASTRA-3	OP648138
Ralstonia insidiosa strain MBL	OP649852
Enterococcus faecalis strain SASTRA-2	OP648136

Table. S3 Strains isolated from Vaginal Swab other than *Lactobacillus* sp. and their accession numbers.

Peak#	*R.Time*	*Area*	*Area%*	*A/H*	*Similarity*	*Name*
1	*14.877*	*80700*	*0.69*	*2.91*	*93*	*Indole*
2	*16.014*	*8841*	*0.08*	*2.04*	*69*	*Quinazoline, 4-methyl-*
3	*16.979*	*142724*	*1.21*	*3.07*	*95*	*Indole, 3-methyl-*
4	*19.507*	*81298*	*0.69*	*2.12*	*92*	*2,4-Di-tert-butylphenol*
5	*20.672*	*44131*	*0.37*	*2.69*	*84*	*Isoquinoline, 3-methyl*
6	*22.719*	*17102*	*0.15*	*2.63*	*74*	*2(3H)-Benzothiazolone*
7	***24.004***	***11148356***	***94.63***	***3.81***	***95***	***Tryptamine***
8	*25.090*	*105949*	*0.90*	*2.70*	*83*	*1H-Indole-3-acetonitrile*
9	*27.036*	*5796*	*0.05*	*1.14*	*63*	*3-Phenyl-2-pentenenitrile*
10	*27.199*	*9561*	*0.08*	*2.00*	*62*	*Octanoic acid, 8-hydroxy-, methyl ester*
11	*27.885*	*15411*	*0.13*	*2.32*	*69*	*9H-Pyrido[3,4-b]indole, 1-methyl-*
12	*28.075*	*120972*	*1.03*	*3.65*	*88*	*9H-Pyrido[3,4-b]indole*

Table. S4 Gas Chromatography-Mass Spectrometry (GC-MS) of Compound 2(C2). The peak analysis shows that the compound is Tryptamine (Indicated in bold)

**Type of Motility**	**Control**	***L. crispatus***		***L. jensenii***		***L. fermentum* NANDAN**	
	(mm)	MIC (4 µl) (mm)	Sub MIC (2 µl) (mm)	MIC (4 µl) (mm)	Sub MIC (2 µl) (mm)	MIC (4 µl) (mm)	Sub MIC (2 µl) (mm)
Swarming	0.167	0.056	0.067	0.033	0.056	0.056	0.070
Swimming	0.0178	0.044	0.033	0.026	0.022	0.044	0.061

Table. S5 CFS treatment affects the Swarming and Swimming Motility of *E. coli* UTI89. The measurements of swarming and swimming motility were performed using ImageJ software. The diameters are measured in millimeters (mm). Each value represents the mean of three replicates (n=3).

**Type of Motility**	**Untreated**	***Lactobacillus* derived tryptamine**		**Commercial Tryptamine**		**CFS**	
	(mm)	MIC (4 µl) (mm)	Sub MIC (2 µl) (mm)	MIC (4 µl) (mm)	Sub MIC (2 µl) (mm)	MIC (4 µl) (mm)	Sub MIC (2 µl) (mm)
Swarming (A)	0.356	0.329	0.321	0.364	0.382	0.080	0.089
Swimming (B)	1.474	1.040	1.153	1.020	1.127	0.104	0.108

Table.S6 Tryptamine treatment affects the Swarming (A) and Swimming(B) Motility of *E. coli* UTI89. Swarming and swimming motility measurements were conducted using ImageJ software. Data represent the mean values from three independent experiments (n=3).

Gene	Gene Product/ Function	Primers (5`-3`)	Temperature, °C	Ref
fimA	Type 1 fimbriae major subunit	ATCGTTGTTCTGTCGGCTCT<br><br>GCGGTACGAACCTGTCCTAA	57	Shivaprasad *et al*, 2021
fim H	Type 1 fimbriae major subunit	GTGCCAATTCCTCTTACCGTT<br><br>*TGGAATAATCGTACCGTTGCG*	64	Hojati *et al*, 2015
csgA	Curli major subunit	GGTAATGGTGCAGATGTTG<br><br>GTCACGTTGACGGAGGAGTT	55	Shivaprasad *et al*, 2021
papG	P-fimbriae tip adhesin	GATTCACCATAGAGGCGACTGC<br><br>GAAATACAACTCCGACATACAGCC	55	Surbhi *et al*, 2021
sfas	S-fimbriae	GTGGATACGACGATTACT GTG<br><br>CCGCCAGCATTCCCTGTA TTC	63	Rana El-baz *et al*, 2022

Table. S7 List of primers used for the Gene Expression Study

Organism	2hr	4hr	6hr	12hr
Limosilactobacillus fermentum strain NANDAN	73.47 ±0.1	74.31±0.03	75.77±0.08	88.67±0.009
Lactobacillus crispatus	33.64 ±0.001	44.2±0.001	47.33±0.003	55.43±0.006
Lactobacillus amylovorus	51.13 ± 0.02	60.31±0.01	64.27±0.015	67.2±0.005
Ligilactobacillus salivarius	4.4± 0.0005	6.8±0.01	21.07±0.09	81.55±0.01
Weissella confusa	56.1± 0.025	60.7±0.02	64.54±0.01	78.6±0.004
Lactobacillus jensenii	21.31±0.0041	32.51±0.0031	59±0.003	73.14±0.0012
Lactobacillus fornicalis	16.13±0.02	29.38±025	36.18±0.013	49.12±0.012
Limosilactobacillus fermentum strain *SASTRA_SSV*	70.13±0.014	72.4±0.04	80.62±0.001	89.31±0.0021
Lactobacillus fermentum MTCC	70.53±0.011	72.21±0.003	69.16±0.021	68.04±0.021
Lactobacillus rhamnosus MTCC	64.32±0.013	68.14±0.004	74.32±0.013	72.41±0.003

Table. S8 Autoaggregation of isolated vaginal *Lactobacilli* at different time points (2,4,6 and 12h respectively). Results are expressed as the average value and standard deviation of three replicates.

Organism	2hr
Limosilactobacillus fermentum strain NANDAN	2±0.4
Lactobacillus crispatus	86.8±0.007
Weissella confusa	68.8±0.05
Lactobacillus amylovorus	52.6±0.3
Ligilactobacillus salivarius	67±0.01
Limosilactobacillus fermentum strain SASTRA_SSV	3.13±0.01
Lactobacillus jensenii	72.41±0.021
Lactobacillus fornicalis	62.13±0.011
Lactobacillus fermentum MTCC	6.23± 0.012
Lactobacillus rhamnosus MTCC	67.45±0.0002

Table. S9 Hydrophobicity of the isolated *Lactobacilli* strains. Results are expressed as the average value and standard deviation of three replicates (n=3)

	Percentage of aggregation				
**Probiotic organisms**	**MDR *Klebsiella oxytoca***	***Klebsiella pneumoniae***	***Citrobacter koserii***	***E. coli* UTI89**	***E. coli CFT073***
Limosilactobacillus fermentum strain NANDAN	50 ± 0.001	59.7 ± 0.012	67.9 ±0.0021	56.2±0.011	53.6 ±0.0021
Lactobacillus crispatus	45.08±0.01	58.7 ±0.12	57.21±0.0213	54 ±0.0021	63.31±0.031
Weissella confusa	39.79±0.0032	68.29±0.0142	37.89±0.012	57±0.019	54.8±0.0001
Lactobacillus amylovorus	54.4±0.011	75.2 ±0.0021	52.6±0.031	62.9±0.05	58.17±0.04
Ligilactobacillus salivarius	53.02±0.0012	68.3±0.04	52.2±0.0051	58.7±0.021	50.2±0.0025
Limosilactobacillus fermentum strain SASTRA_SSV	53.13±0.13	56.12±0.014	68.42±0.015	61.14±0.0021	72.16±0.012
Lactobacillus jensenii	52.41±0.001	58.32±0.003	61.36±0.0021	59.47±0.013	69.13±0.0001
Lactobacillus fornicalis	52.13±0.001	69.18±005	56.38±0.003	69.22±0.012	61.03±0.021
Lactobacillus fermentum MTCC	74.53±0.0021	72.21±0.003	69.16±0.021	68.04±0.021	71.02±0.001
Lactobacillus rhamnosus MTCC	64.32±0.03	70.24±0.015	64.32±0.003	62.41±0.023	69.21±0.021

Table.S10 Coaggregation of isolated vaginal *Lactobacillus* strains with clinically relevant uropathogens. Results are expressed as average value and standard deviation of replicates (n= 3)

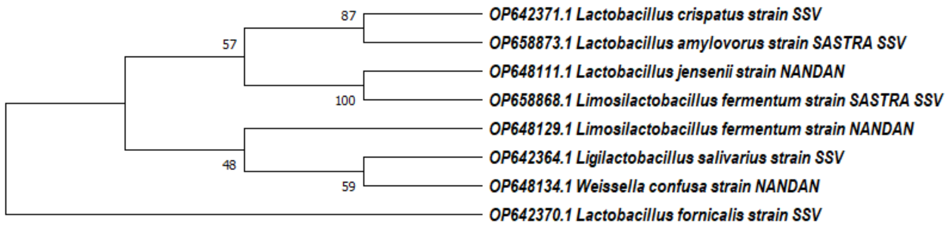

Fig. S1 Phylogenetic tree of isolated *Lactobacilli* sps. Constructed using MEGA 11 software boot strap method.

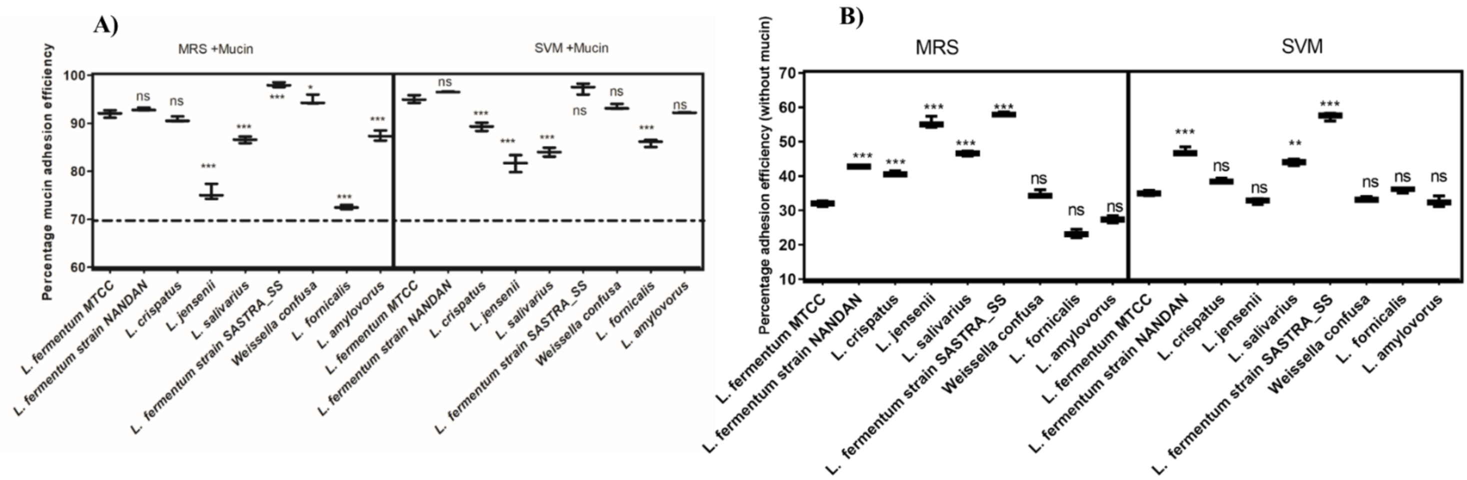

Fig. S2 Adhesion capacity of the isolated *Lactobacillus* strains in the presence and absence of Mucin. The dotted line indicates the range of the adhesion is >70% **A)** Presence of Mucin **B)** Absence of Mucin. Box and whiskers plot with one-way analysis of variance, p<0.005 (***) versus control (*L. fermentum* MTCC)

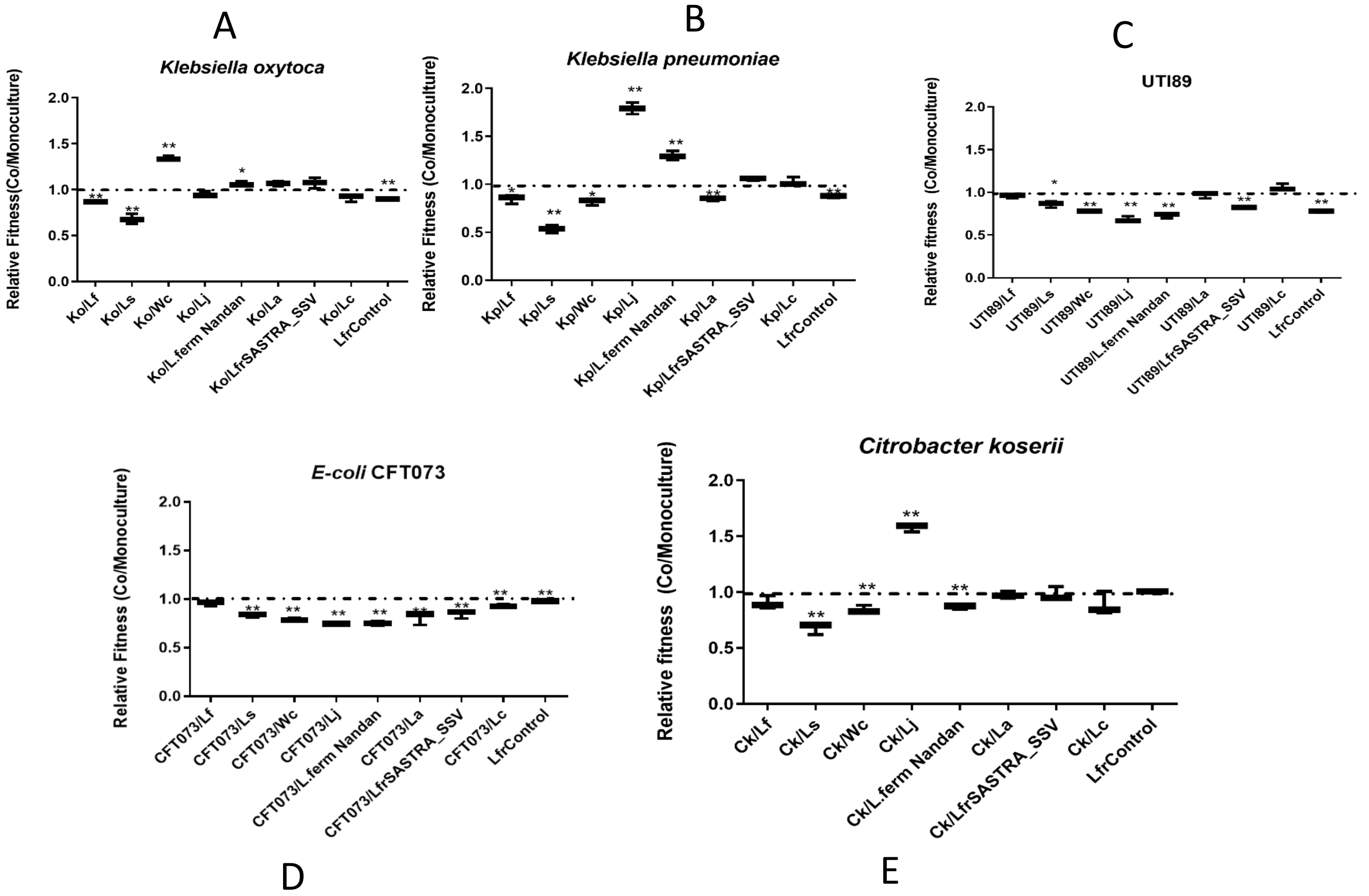

Fig. S3 Competitive adhesion of Vaginal Lactobacilli sp. against the uropathogens. Lactobacilli were competed with A) *Klebsiella oxytoca* B) *Klebsiella pneumoniae* C) *E-coli* CFT073 D) *E-coli* UTI89 E) *Citrobacter koserii*. n=3, One-tail t test was performed to determine the significance, * $p<0.05$, **$p<0.01$, ***$p<0.001$. and the Relative Fitness (RM) values are calculated as the ratio of the pathogen to the Lactobacillus sp. isolate. Values >1 indicate higher growth of the pathogenic strain and <1 indicate higher growth of the *Lactobacilli* sp. in the co-culture conditions.Lf - *L. fornicalis*, Ls-*L. salivarius*, Wc- *W. confusa*, Lj- *L. jensenii*, LfrNANDAN- *L. fermentum* strain NANDAN, LfrSASTRA_SSV- *L. fermentum* SASTRA_SSV, La- *L. amylovorus*, Lc -*L. crispatus*, Lfr- *L. fermentum* control (MTCC), Ck- *C. koserii*, Kp-*K. pneumoniae*, Ko- *K. oxytoca*, UTI89- *E-coli* UTI89, CFT073- *E-coli* CFT073.

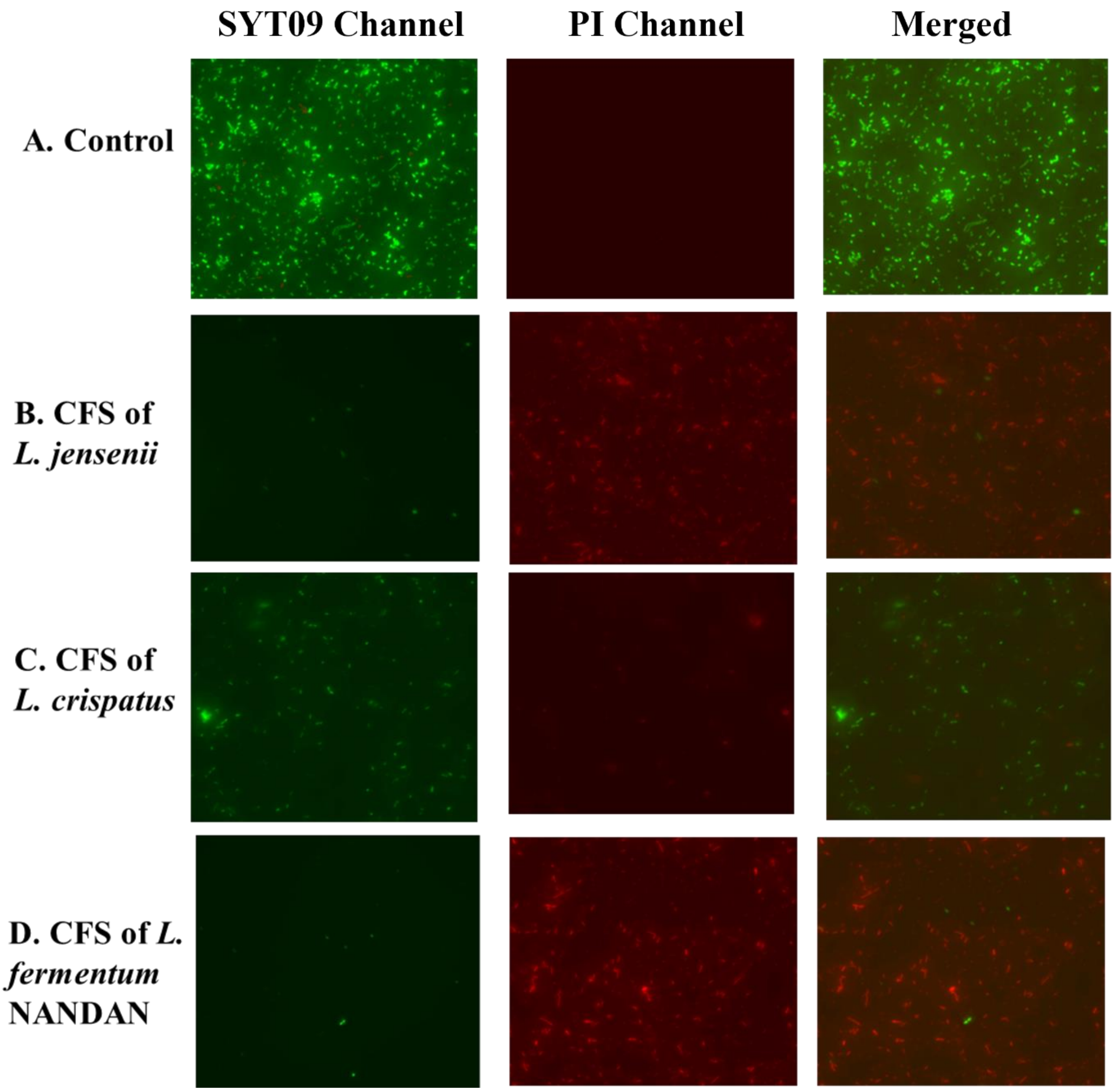

Fig. S4 Live/Dead stain performed after treating *E. coli* biofilm with CFS of *L. jensenii*, *L. crispatus* and *L. fermentum* NANDAN. A) Control B) CFS of *L. jensenii* C) CFS of *L crispatus* D) CFS of *L. fermentum* NANDAN. All images have the scale of 20 µm.

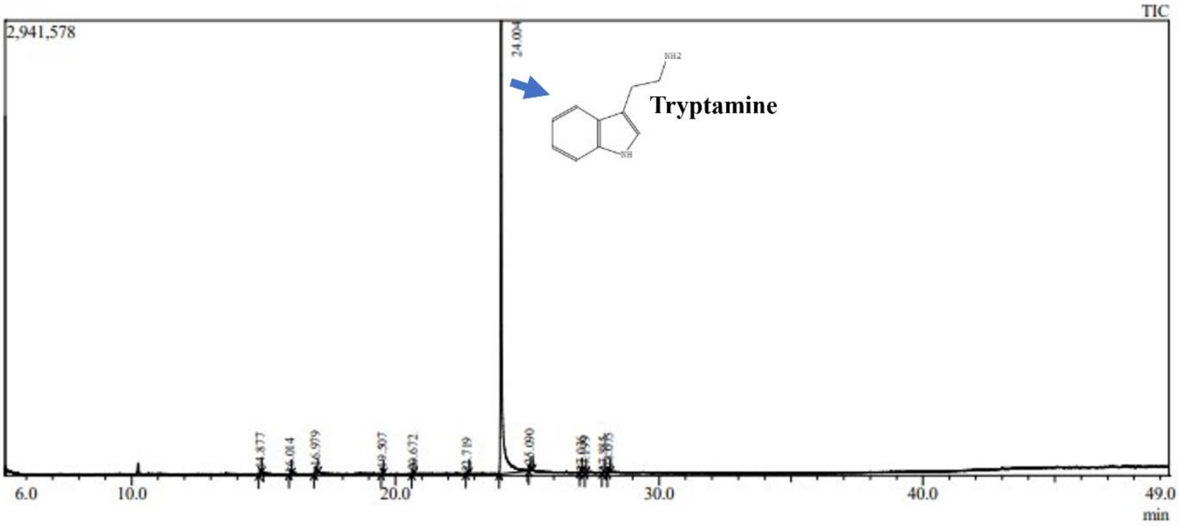

Fig. S5 Gas Chromatography-Mass Spectrometry (GC-MS) of Compound 2(C2). The highest peak with retention time 24.004 displayed 95% similarity to Tryptamine spectra in National Institute of Standards and Technology (NIST) 20 database.

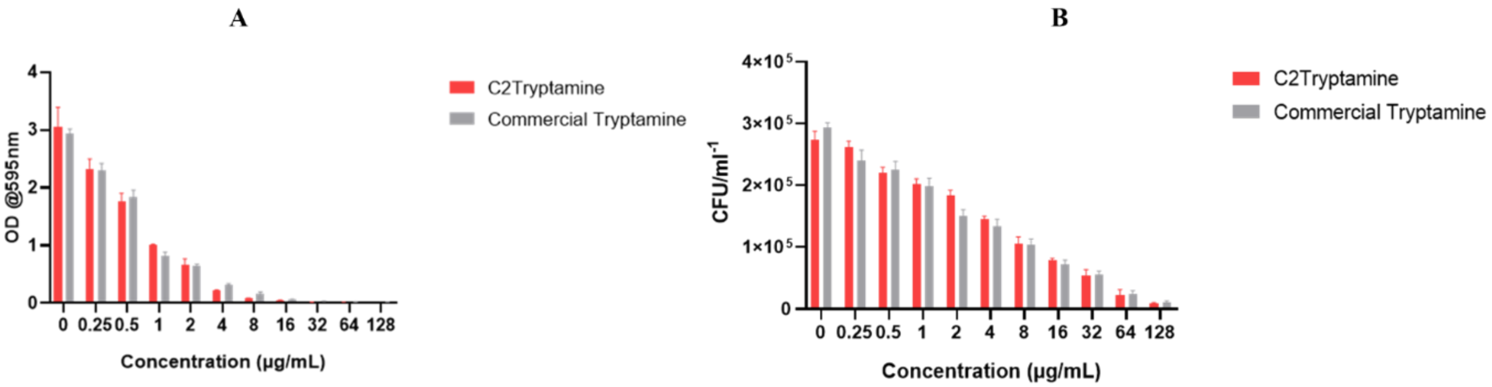

Fig. S6 A) Minimum biofilm eradication concentration (MBEC) and B) Minimum Inhibition Concentration (MIC) and of both purified Tryptamine from CFS and Commercial tryptamine

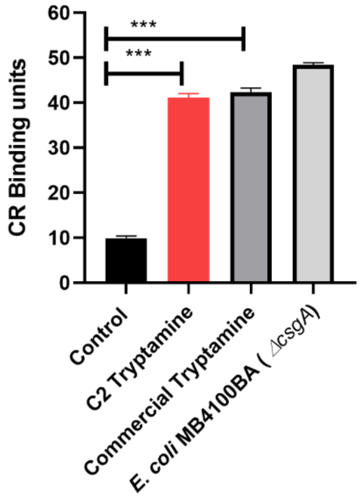

Fig. S7 Quantification of curli production by congored binding assay, curli deficient mutant *E. coli* MB4100BA strain was used as the negative control. Unpaired t-test was used to determine the significance ($P < 0.001$), $n = 3$

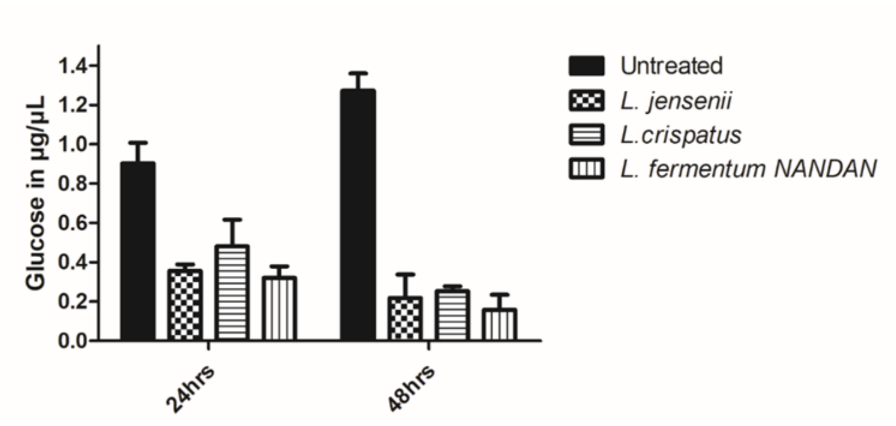

Fig. S8 The impact of cell-free supernatant (CFS) derived from three *Lactobacillus* species, (*Lactobacillus jensenii, Lactobacillus crispatus, and Lactobacillus fermentum)* on extracellular polymeric substances (EPS) production. EPS quantification was performed at 24 and 48 hours of biofilm growth.

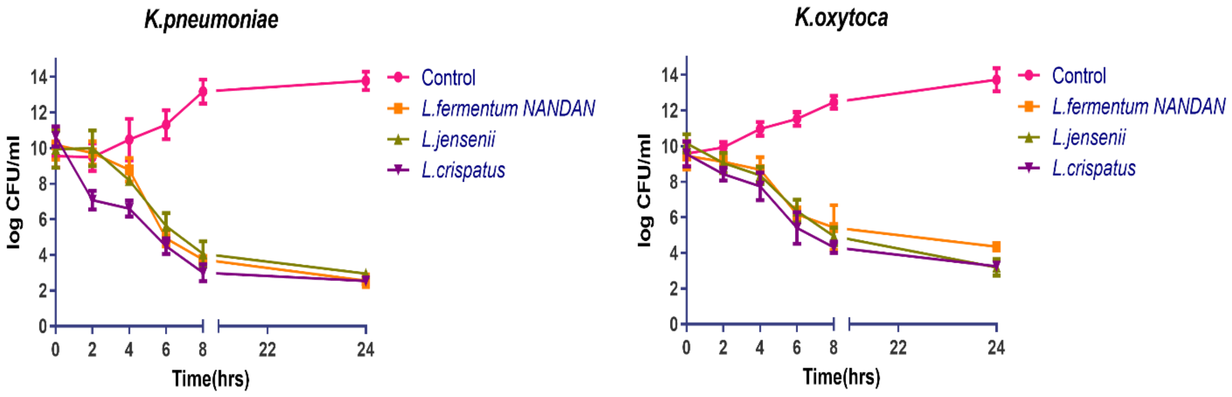

A B

Fig. S9 Time-kill assay evaluating the efficacy of cell-free supernatants from three selected *Lactobacilli* strains (*L. fermentum NANDAN, L. crispatus, and L. jensenii*) against biofilm derived cells of uropathogens. A) *K. pneumoniae* B) MDR *K. oxytoca.* The error bars represent the standard deviation of three replicates performed.

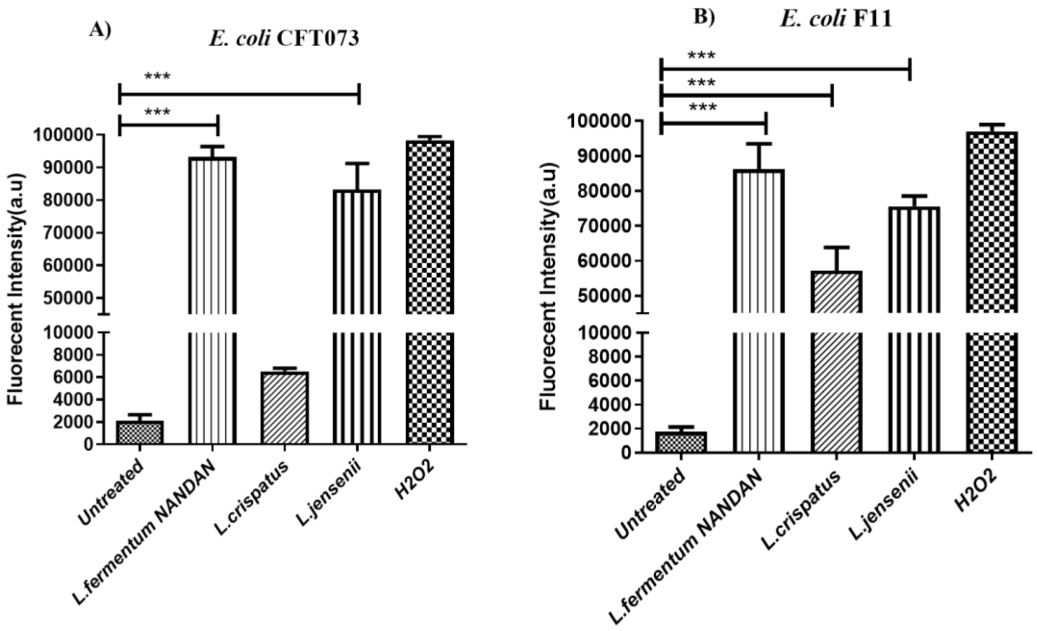

Fig. S10 Reactive Oxygen Species (ROS) Generation in Uropathogenic Biofilms Treated with CFS A) *E. coli* CFT073 B) *E. coli* F11. The experimental group treated with CFS exhibited a significant increase in ROS generation compared to the control group. Statistical significance was determined using Student's t-test (***<0.001).

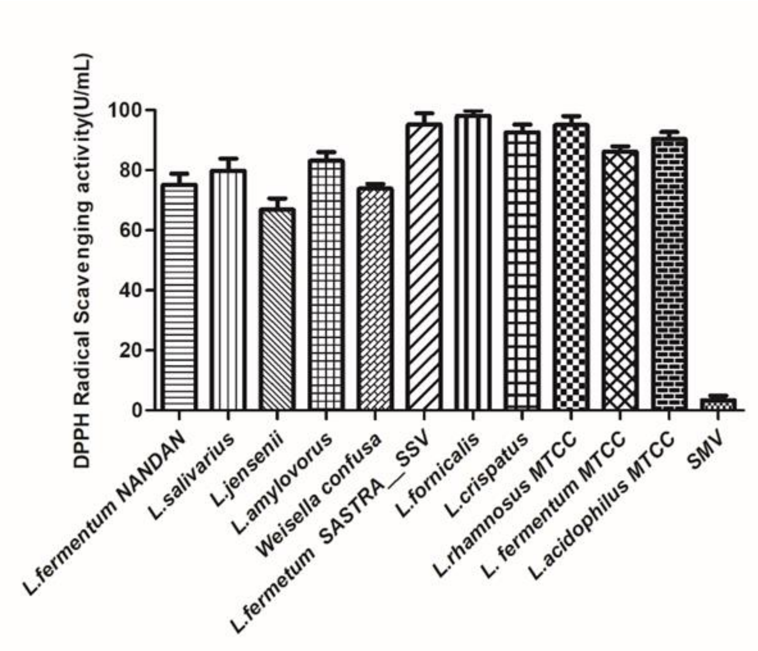

Fig. S11 Antioxidant Property of the isolated *Lactobacillus sp***.** DPPH activity of isolated Lactobacillus sp. (mean ± SD, n = 3), Simulated Vaginal Media (SMV) without organism is used as the negative control and *L. rhamnosus* MTCC, *L. fermentum* MTCC and *L. acidophilus* MTCC were used as positive control.

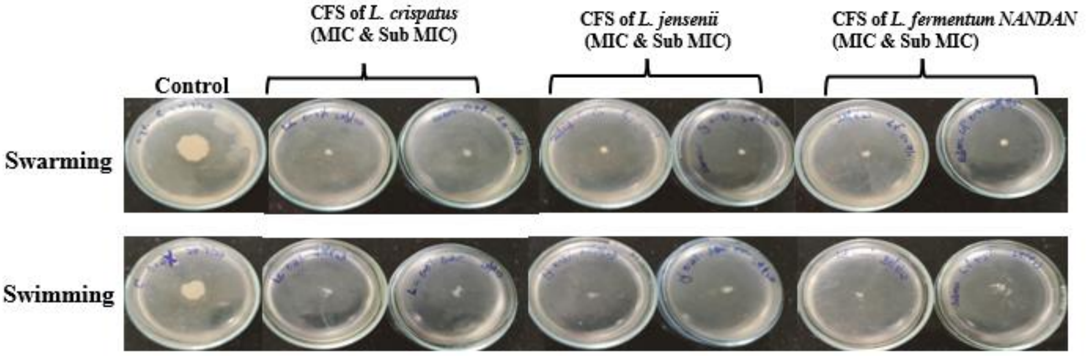

Fig. S12 CFS treatment affects the Swarming and Swimming Motility of *E. coli* UTI89. Images of Swarming and Swimming motility. Motility assays was performed with *E. coli* UTI89. 2 µl of midlog phase bacteria were spotted on 0.7%and 0.3% soft agar for swarming and swimming respectively which was incorporated with either MIC or Sub MIC levels of CFS (*L. crispatus, L. jensenii and L. fermentum NANDAN*)

A)

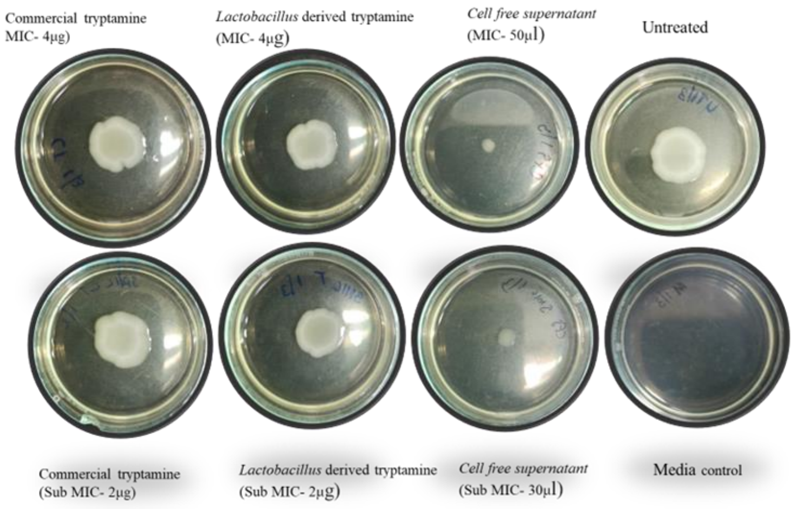

B)

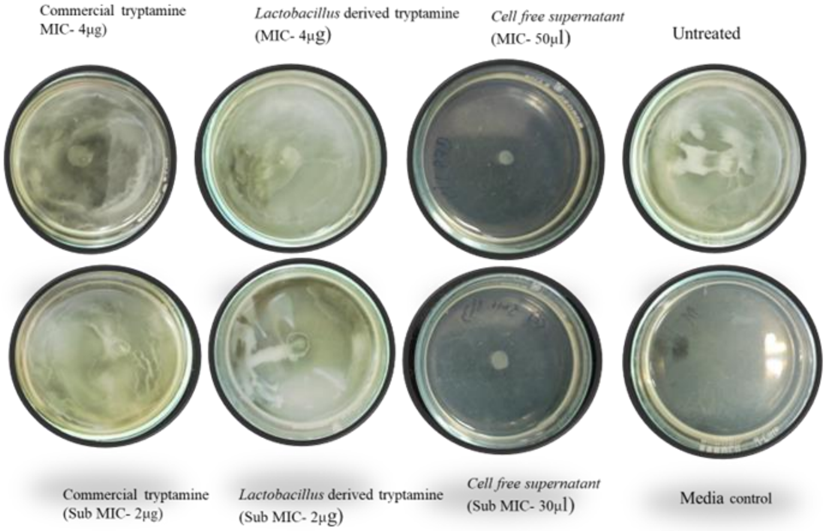

Fig. S13 Effect of Tryptamine treatment on Swarming (A) and Swimming(B) Motility of *E. coli* UTI89. Images of Swarming and Swimming motility. Motility assays was performed with *E. coli* UTI89. 2 µl of midlog phase bacteria were spotted on 0.7%and 0.3% soft agar for swarming and swimming respectively which was incorporated with either MIC or Sub MIC levels of Lactobacillus derived and procured tryptamine. Untreated is used as a control.

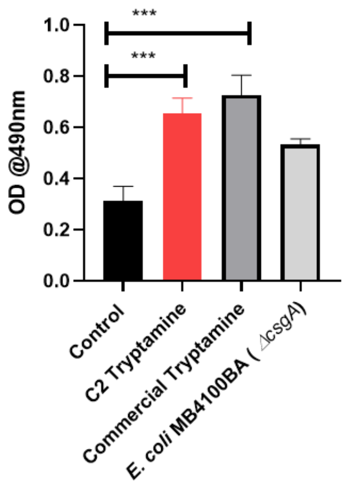

Fig. S14 Matrix quantification by Congored depletion assay: Absorbance values after treatment with 8µg/ml of tryptamine, indicating the lowest observed matrix production in the study. Unpaired t-test, n= 3, 95% CI, $p^{***} < 0.0001$

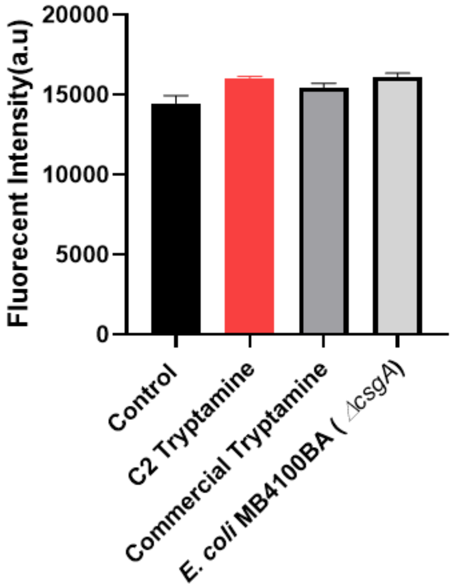

Fig. S15 Cellulose quantification using Calcofluor staining**.** Calcofluor only binds to the cellulose. The graph shows there is no decrease in the cellulose of *E. coli* biofilm matrix after treating with tryptamine. The positive control used was ∆csgA E. coli MB4100BA.

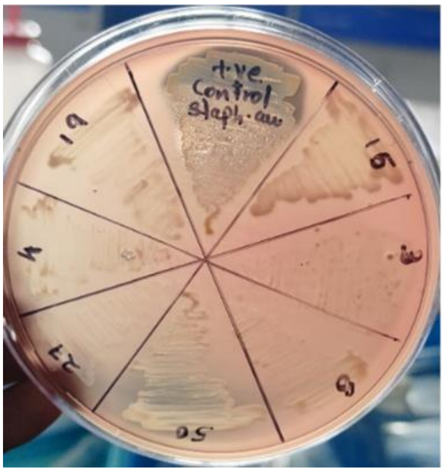

Fig. S16 Haemolytic activities of isolated *Lactobacillus* were evaluated on blood agar plates. None of the tested strains showed haemolysis on blood agar plates. Positive control used is Staphylococcus aureus ATCC 25923

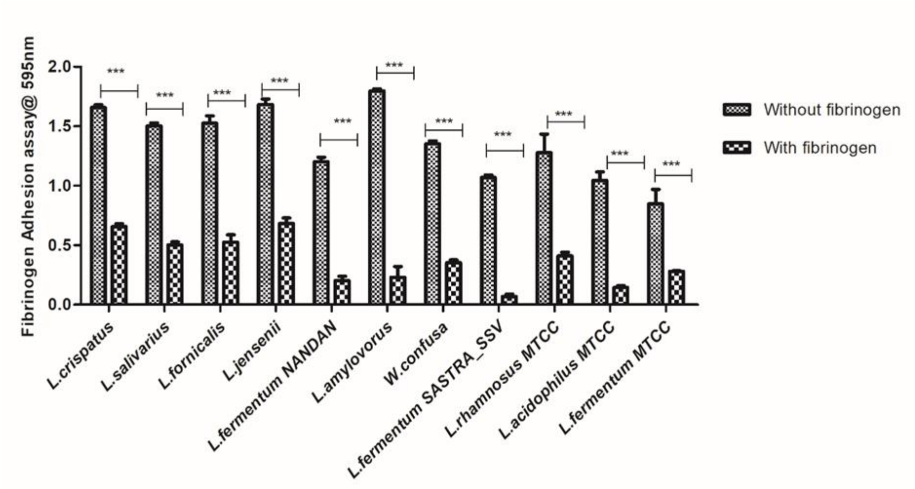

Fig. S17 Fibrinogen Adhesion assay of isolated *Lactobacillus* sp**.** One sample *t*-test had done and the significant difference ($p<0.0001$***) was calculated after comparing with and without fibrinogen.

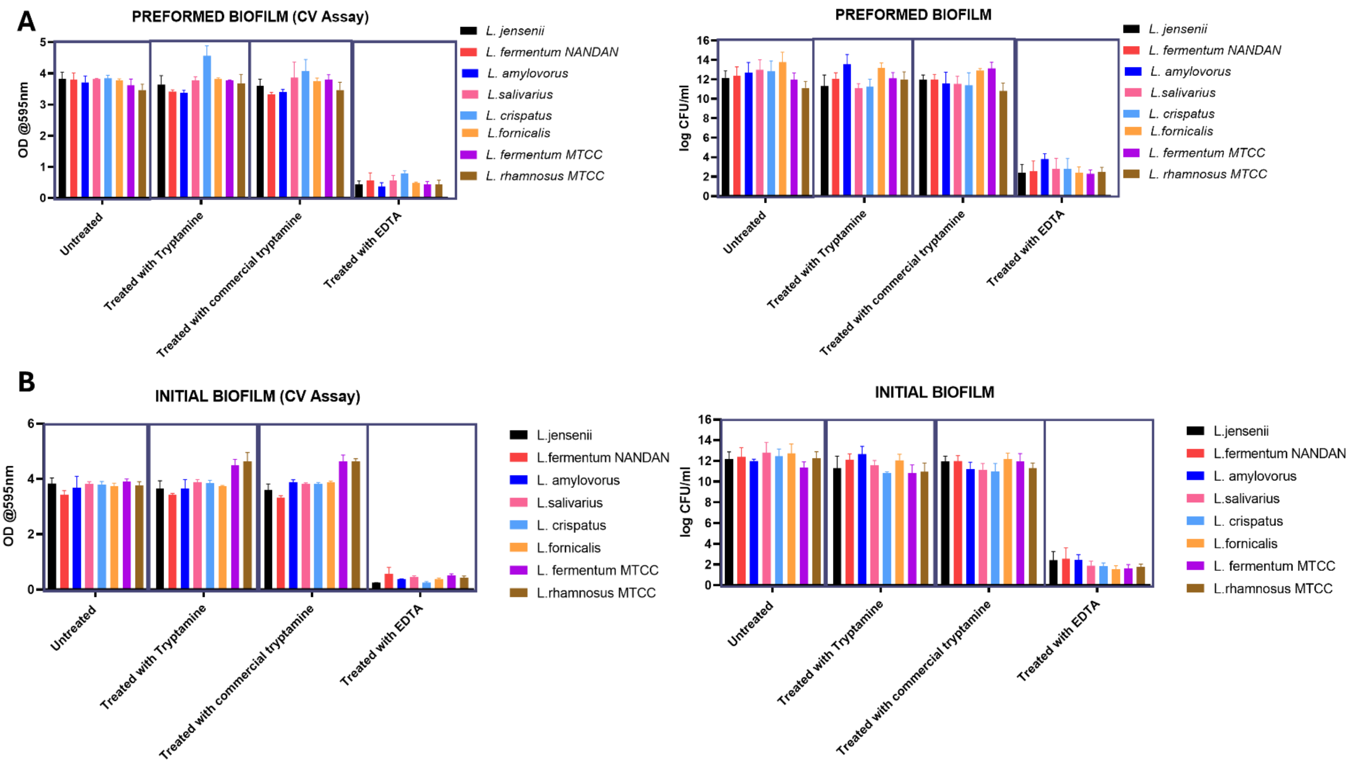

Fig S18: Tryptamine's impact on initial and preformed *Lactobacillus* Biofilms. A) Preformed *Lactobacillus* biofilms treated with tryptamine (8 µg/mL) compared to untreated controls. The biofilms treated with tryptamine maintained similar structural integrity as the untreated biofilms, demonstrating that tryptamine does not affect the preformed *Lactobacillus* biofilms. B) Growth and biofilm formation of *Lactobacillus* strains (initial biofilms) treated with tryptamine compared to untreated controls. No significant inhibition of growth or biofilm formation was observed, indicating that tryptamine does not adversely affect *Lactobacillus* biofilm formation. Positive control using EDTA (0.1%), which significantly distorted the *Lactobacillus* biofilm. Error bars represent the standard deviation from three independent experiments.
